# Supplementary material for: Impact of point-of-care panel tests in ambulatory care: a systematic review and meta-analysis
Source: BMJ Open. 2020 Feb 27;10(2):e032132. doi: 10.1136/bmjopen-2019-032132 (PMC7050348; doi:10.1136/bmjopen-2019-032132)

Appendix 1: Search Strategy

1	Ambulatory Care/
2	exp Ambulatory Care Facilities/
3	general practice/ or family practice/
4	general practitioners/ or physicians, family/ or physicians, primary care/
5	Primary Health Care/
6	Office Visits/
7	exp Emergency Service, Hospital/
8	Emergency Medical Services/
9	(ambulatory adj3 (care or setting? or facilit* or ward? or department? or service?)).ti,ab.
10	((general or family) adj2 (practi* or physician? or doctor?)).ti,ab.
11	(primary care or primary health care or primary healthcare).ti,ab.
12	(emergency adj3 (care or setting? or facilit* or ward? or department? or service?)).ti,ab.
13	(after hour? or afterhour? or "out of hour?" or ooh).ti,ab.
14	(clinic? or visit?).ti,ab.
15	((health* or medical) adj2 (center? or centre?)).ti,ab.
16	community health services/ or exp community health nursing/
17	Community Health Workers/
18	(community adj2 (health or health care or service? or program*)).ti,ab.
19	(community adj2 (worker? or aide? or volunteer? or assistant? or visitor?)).ti,ab.
20	((lay or volunteer) adj2 (health worker? or health aide? or health assistant?)).ti,ab.
21	((health* or medical) adj2 (facility or facilities)).ti,ab.
22	1 or 2 or 3 or 4 or 5 or 6 or 7 or 8 or 9 or 10 or 11 or 12 or 13 or 14 or 15
23	16 or 17 or 18 or 19 or 20 or 21
24	Point-of-Care Systems/
25	(("point of care" or POC) adj3 (test* or diagnos*)).ti,ab.
26	(("point of care" or POC) and (test* or diagnos*)).ti.

27	poct.ti,ab.
28	((rapid or bedside or bed-side or "near patient") adj3 (test* or diagnos*)).ti,ab.
29	((rapid or bedside or bed-side or "near patient") and (test* or diagnos*)).ti.
30	24 or 25 or 26 or 27 or 28 or 29
31	(istat or i-stat or afinion).ti,ab.
32	30 or 31
33	22 and 32
34	23 and 32
35	34 not 33

Appendix 2: Risk of bias assessment for Parvin et al, before-after study[27]

Selection		Performance	Detection	Attrition	Reporting	Other	Pre-specified confounders		
Random sequence generation	Allocation concealment	Blinding or participants/ personnel	Blinding of outcome assessment	Incomplete outcome data	Selective reporting		Baseline charactertisitics reported	Baseline charactersitic similar in intervention and control groups	Detailed description of usual care pathway
not randomised<br><br>**HIGH RISK**	not concealed; time periods of intervention and control described<br><br>**HIGH RISK**	none<br><br>**HIGH RISK**	not described<br><br>**UNCLEAR RISK**	not described<br><br>**UNCLEAR RISK**	not described<br><br>**UNCLEAR RISK**	control groups from before and after the intervention period; retrospective designation of presenting symptom codes<br><br>**HIGH RISK**	not reported<br><br>**HIGH RISK**	not reported<br><br>**HIGH RISK**	no details other than that samples are sent to central laboratoy via vacuum transport system<br><br>**HIGH RISK**

Appendix 3

Sensitivity Analysis

Figure 3: Disposition Decision (subgroups for patients who needed laboratory tests in addition to POCT)

Figure 3a) With Illahi[25]

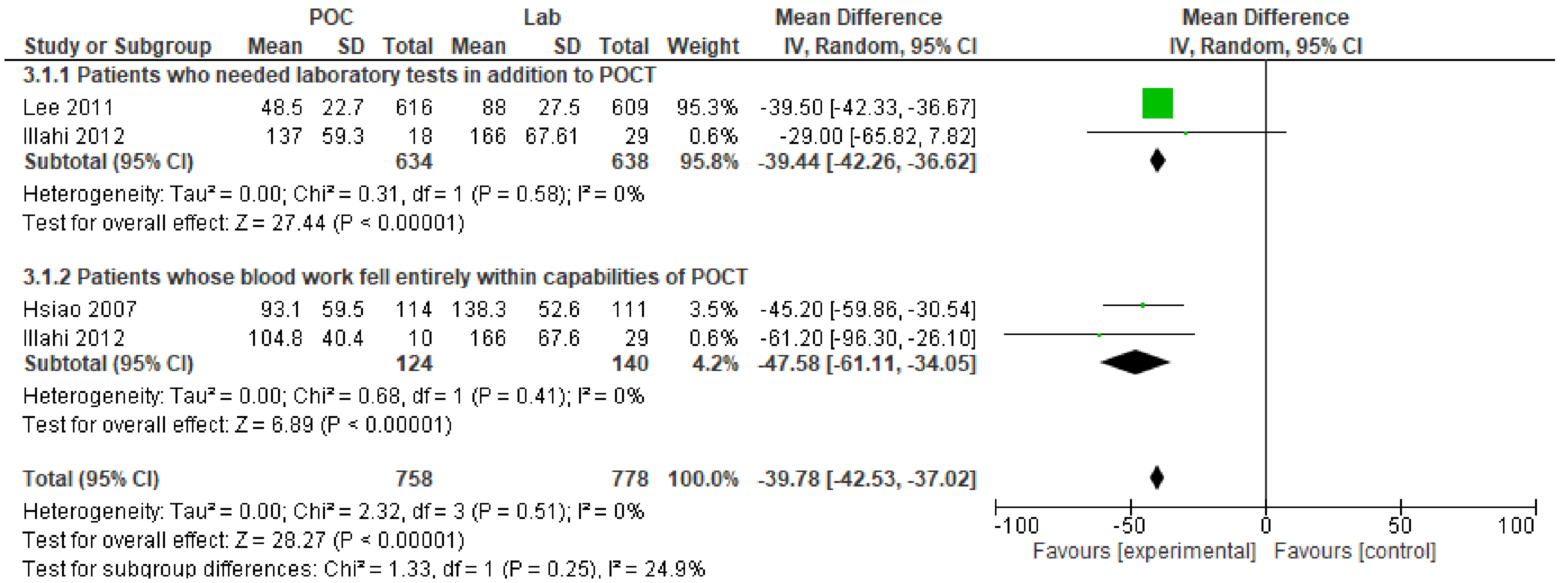

Figure 3b) Without Illahi[25]

	POC			Lab				Mean Difference
Study or Subgroup	Mean	SD	Total	Mean	SD	Total	Weight	IV, Random, 95% CI
**3.1.1 Patients who needed laboratory tests in addition to POCT**								
Lee 2011	48.5	22.7	616	88	27.5	609	96.4%	-39.50 [-42.33, -36.67]
**Subtotal (95% CI)**			**616**			**609**	**96.4%**	**-39.50 [-42.33, -36.67]**
Heterogeneity: Not applicable								
Test for overall effect: Z = 27.40 (P < 0.00001)								
**3.1.2 Patients whose blood work fell entirely within capabilities of POCT**								
Hsiao 2007	93.1	59.5	114	138.3	52.6	111	3.6%	-45.20 [-59.86, -30.54]
**Subtotal (95% CI)**			**114**			**111**	**3.6%**	**-45.20 [-59.86, -30.54]**
Heterogeneity: Not applicable								
Test for overall effect: Z = 6.04 (P < 0.00001)								
**Total (95% CI)**			**730**			**720**	**100.0%**	**-39.70 [-42.48, -36.93]**
Heterogeneity: Tau² = 0.00; Chi² = 0.56, df = 1 (P = 0.45); I² = 0%								
Test for overall effect: Z = 28.05 (P < 0.00001)								
Test for subgroup differences: Chi² = 0.56, df = 1 (P = 0.45), I² = 0%								

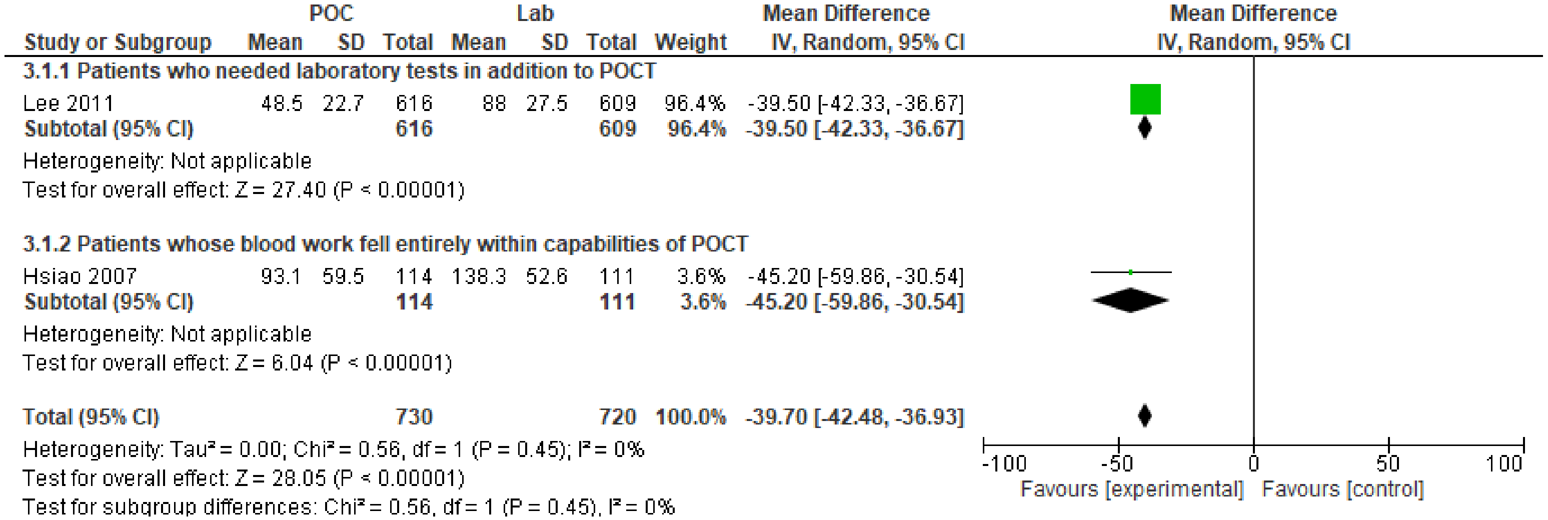

Supplement: Supplementary data [file bmjopen-2019-032132supp001.pdf]
