# Supplementary material for: Impact of point-of-care panel tests in ambulatory care: a systematic review and meta-analysis
Source: BMJ Open. 2020 Feb 27;10(2):e032132. doi: 10.1136/bmjopen-2019-032132 (PMC7050348; doi:10.1136/bmjopen-2019-032132)
Supplement: Author's manuscript [file bmjopen-2019-032132.draft_revisions.pdf]

BMJ Open is committed to open peer review. As part of this commitment we make the peer review history of every article we publish publicly available.

When an article is published we post the peer reviewers' comments and the authors' responses online. We also post the versions of the paper that were used during peer review. These are the versions that the peer review comments apply to.

The versions of the paper that follow are the versions that were submitted during the peer review process. They are not the versions of record or the final published versions. They should not be cited or distributed as the published version of this manuscript.

BMJ Open is an open access journal and the full, final, typeset and author-corrected version of record of the manuscript is available on our site with no access controls, subscription charges or pay-per-view fees (http://bmjopen.bmj.com).

If you have any questions on BMJ Open's open peer review process please email info.bmjopen@bmj.com

BMJ Open

Impact of point-of-care panel tests in ambulatory care: a systematic review and meta-analysis

Journal:	*BMJ Open*
Manuscript ID	bmjopen-2019-032132
Article Type:	Research
Date Submitted by the Author:	04-Jun-2019

Complete List of Authors:	Goyder, Clare; University of Oxford, Nuffield Department of Primary Care Health Sciences<br>Tan, Pui San; University of Oxford, Nuffield Department of Primary Care Health Sciences<br>Verbakel, Jan; University of Oxford, Nuffield Department of Primary Care Health Sciences; KU Leuven, Department of Public Health and Primary Care<br>Ananthakumar, Thanusha; University of Oxford, Nuffield Department of Primary Care Health Sciences<br>Lee, Joseph; University of Oxford, Nuffield Department of Primary Care Health Sciences<br>Hayward, Gail; University of Oxford, Nuffield Department of Primary Care Health Sciences<br>Turner, Philip; University of Oxford, Nuffield Department of Primary Care Health Sciences<br>van den Bruel, Ann; University of Oxford, Department of Primary Care Health Sciences

Keywords:	point of care tests, POCT, ACCIDENT & EMERGENCY MEDICINE, PRIMARY CARE, Paediatric A&E and ambulatory care < PAEDIATRICS

SCHOLARONE™
Manuscripts

I, the Submitting Author has the right to grant and does grant on behalf of all authors of the Work (as defined in the below author licence), an exclusive licence and/or a non-exclusive licence for contributions from authors who are: i) UK Crown employees; ii) where BMJ has agreed a CC-BY licence shall apply, and/or iii) in accordance with the terms applicable for US Federal Government officers or employees acting as part of their official duties; on a worldwide, perpetual, irrevocable, royalty-free basis to BMJ Publishing Group Ltd ("BMJ") its licensees and where the relevant Journal is co-owned by BMJ to the co-owners of the Journal, to publish the Work in this journal and any other BMJ products and to exploit all rights, as set out in our licence.

The Submitting Author accepts and understands that any supply made under these terms is made by BMJ to the Submitting Author unless you are acting as an employee on behalf of your employer or a postgraduate student of an affiliated institution which is paying any applicable article publishing charge ("APC") for Open Access articles. Where the Submitting Author wishes to make the Work available on an Open Access basis (and intends to pay the relevant APC), the terms of reuse of such Open Access shall be governed by a Creative Commons licence – details of these licences and which Creative Commons licence will apply to this Work are set out in our licence referred to above.

Other than as permitted in any relevant BMJ Author's Self Archiving Policies, I confirm this Work has not been accepted for publication elsewhere, is not being considered for publication elsewhere and does not duplicate material already published. I confirm all authors consent to publication of this Work and authorise the granting of this licence.

For peer review only

Impact of point-of-care panel tests in ambulatory care: a systematic review and meta-analysis

Clare R Goyder, GP/Clinical Researcher[1]
Pui San Tan, Researcher[1]
Jan Y Verbakel, GP/Assistant professor[1,2]
Thanusha Ananthakumar, GP/Clinical Researcher[1]
Joseph Lee, GP/Clinical Researcher[1]
Gail Hayward, GP/Academic Clinical Lecturer[1]
Philip Turner, Researcher[1]
Ann Van den Bruel, GP/Associate professor[1]

1. Nuffield Department of Primary Care Health Sciences, University of Oxford, Oxford OX2 6GG, UK

2. Department of Public Health and Primary Care, KU Leuven
Kapucijnenvoer 33, 3000 Leuven, Belgium

Corresponding author: clare.goyder@phc.ox.ac.uk

For peer review only - http://bmjopen.bmj.com/site/about/guidelines.xhtml

Abstract

Objective

This article summarizes all the available evidence on the impact of introducing point-of-care panel testing in ambulatory care on patient outcomes and healthcare processes.

Methods

This systematic review and meta-analysis included randomised controlled trials (RCTs) and before-after studies in ambulatory care. Medline, Embase, Cochrane Database of Systematic Reviews and Cochrane Central were systemically searched. The primary outcome was the time to disposition decision. Secondary outcomes included length of stay and mortality.

Results 19,562 patients from nine studies were included in the review, eight of these were RCTs, and one was a before-after study. All the studies were based in either emergency departments or the ambulance service. General panel tests performed at the point-of-care resulted in disposition decisions being made 40 minutes faster (95% CI -43 to -37, I^2=0%) compared to the usual care group. This in turn resulted in a reduction in length of stay for patients who were subsequently discharged by 34 minutes (95% CI -64 to -5). No significant difference in mortality was reported.

Discussion: Our results suggest that point-of-care panel tests might be most useful in settings where a substantial proportion of patients will not require hospital admission after limited diagnostic assessment, and may lead to faster discharge decisions and a shorter length of stay. Future research should also be performed in primary care and identify how point-of-care tests can contribute meaningful changes to patient care rather than focussing on health care processes and should also consider the patients perspective.

Systematic review registration PROSPERO CRD42016035426

For peer review only - http://bmjopen.bmj.com/site/about/guidelines.xhtml

Strengths and limitations of this study

- This study was conducted robustly, following Preferred Reporting Items for Systematic Reviews and Meta-analyses guidelines using a comprehensive search strategy in the major medical databases, and selection and quality assessment performed by two independent reviewers
- It offers results on the impact of panel point-of-care tests, rather than just their accuracy
- Included studies were relatively small, and most notably for mortality, the results may suffer from power problems
- Statistical and clinical heterogeneity is evident within our meta-analysis, however the meta-analysis was considered carefully and reduced where possible by ensuring that studies of cardiac and general panels tests were not combined; moreover, we did not combine the before-after study (which also had a high risk of bias) with RCTs
- Four studies excluded critically ill patients and patients with myocardial infarction, which may have biased mortality data.

For peer review only

Introduction

Background

All ambulatory care physicians frequently encounter diagnostic uncertainties in day to day practice. This can lead to missed opportunities for diagnoses or inappropriate referrals to secondary care. Patients with vague or non-specific symptoms can be the most challenging populations to assess.[1] Currently, most ambulatory care units use whole blood tests that are normally transported to and processed by a centralised clinical laboratory.

The technology behind in-vitro point-of-care testing (POCT) has developed extensively and the accuracy compared to standard methods for some tests is now established.[2,3] POCT now offers an alternative to conventional laboratory methods; it is performed on site, normally at the bedside and has a short turnaround time of typically 5-15 min.[4] POCT is being employed in a wide variety of healthcare settings and its use is predicted to expand dramatically.[5] Indeed, NHS England have stated that point-of-care tests will be available in urgent treatment centres in the UK from 2019.[6]

Importance

The use of POCT in ambulatory care has the potential to reduce diagnostic uncertainty and delay and physicians report that they would like to use these tests more, particularly to aid in the diagnosis of acute conditions.[7-9] It is expected that POCT facilitates either the speed of discharge, leading to better use of healthcare resources, or enables quicker diagnosis and referral of patients with serious illness, which may lead to better patient outcomes. Panel tests are especially appealing in this patient group as they test multiple parameters simultaneously from the same finger prick of blood using the same platform, covering a range of conditions frequently found to cause acute presentations to ambulatory care. However, there are potential disadvantages associated with their implementation[10] and little is understood about the impact of POCT panels on day to day practice. Thus far, what is lacking is an up to date summary of all the available evidence on the impact of blood based POCT panel testing in ambulatory care.

Objective

In this study, we performed a systematic review and meta-analysis to evaluate the quantitative impact of POCT in ambulatory care with a focus on blood based panel tests.

Methods

This systematic review protocol has been registered in the PROSPERO International Prospective Register of Systematic Reviews (registration number: CRD42016035426). This protocol has been developed according to recommendations from the Cochrane Collaboration[11] and guidelines from the Preferred Reporting Items for Systematic Reviews and Meta-analyses statement[12] have also been followed.

This systematic review forms part of a series of analyses from a larger overall review (in progress) which will assess the overall quantitative impact of all POCTs in ambulatory care, further subgroup analyses are on CRP (awaiting publication)[13] and influenza.[14]

Patient and Public Involvement

We have consulted with an existing PPI panel of the NIHR Diagnostic Evidence Co-operative (DEC) Oxford specialising in research on in-vitro diagnostic technology, who have been involved in a number of previous projects which incorporated POCT. They felt that this systematic review would be very important in

For peer review only

For peer review only - http://bmjopen.bmj.com/site/about/guidelines.xhtml

defining the evidence for and against use of POCT in ambulatory care. One member described her experience as a patient in another European country where POCT for certain conditions was seen as part of standard care, and her surprise that this was not the case in the UK. They were specifically interested in the potential implications of POCT for facilitating earlier discharge from hospital.

Search strategy

We searched Ovid Medline (1946 to 2017), Embase (1974 to 2017), Cochrane Database of Systematic Reviews, Cochrane CENTRAL, DARE, Science Citation Index (1945-present) from the beginning of each database to 2017. The search was originally performed on 19th November 2015 and then updated on 21st March 2017. A snowballing strategy was used to ensure that the search was as comprehensive as possible. We did not add a study design filter nor apply a language restriction. We performed citation searches of all full-text paper included in final review. The full strategy is included in the appendix.

Selection of Studies

We included randomised controlled trials (RCTs), non-randomised but experimental and controlled studies including before after studies. Included studies compared the use of POCT with laboratory testing and were based in ambulatory care.

Screening was divided between six authors (CG, PST, JV, TA, JL, PT); the potential relevance of all titles and abstracts identified from the electronic search were independently assessed by two of these authors. Full-text papers of all potentially relevant papers were obtained and these were then further assessed by two of the authors. Conflicts were resolved by seeking the opinion of a third author and disagreements were discussed with the team to obtain consensus. The reason for excluding studies was recorded. We excluded qualitative studies, conference abstracts, studies focussing on only diagnostic accuracy (comparing the point of care test to a reference standard in a real-life setting), studies that did not have a control group and those evaluating POCT exclusively for monitoring purposes. Studies that included panel tests as part of a multifaceted intervention or combined blood based panel tests with single tests or urine tests were also excluded. Systematic reviews were excluded with reference lists checked for potentially relevant studies for inclusion. For this subgroup analysis, appropriate studies were selected independently by two researchers.

Data extraction and quality assessment

Data extraction was performed by one author and independently checked by a second author. The authors extracted the following data from included studies: general study information (authors, title, publication year, study design and location/setting), inclusion and exclusion criteria and further information regarding the study population (to include mean age and severity of illness of participants), details of the POCT intervention including which parameters were measured by the POCT device, details of the comparator which was normally conventional blood test sent to laboratory; and finally outcomes assessed as listed below.

The methodological quality of the included trials was assessed by two authors (AVB and CG and independently checked by TA). Any areas of conflict were discussed and resolved with a third member of the team where necessary. For RCTs we used the Cochrane Risk of Bias tool[11] including analysis of randomisation, allocation concealment, comparison of baseline characteristics and blinding. For non-randomised but experimental and controlled studies we used the Cochrane Risk of Bias tool plus an assessment of confounders[15] that were pre-specified and included assessing whether baseline characteristics were reported, whether they were similar in intervention and control groups and whether there was a detailed description of the usual care pathway.

For peer review only - http://bmjopen.bmj.com/site/about/guidelines.xhtml

Outcomes assessment

Our priority was to assess the impact of panel based POCT on patient outcomes and healthcare processes. The primary outcome of interest was the impact of POCT on the time to decision regarding disposition i.e admission/referral termed disposition time (DD). Secondary outcomes included length of stay (LOS) at the ambulatory care unit/practice and mortality. Hospital admission rates, rates of repeat attendance after discharge / re-admission were also examined.

Statistical analyses

Individual study estimates were pooled in a meta-analysis using random-effects inverse-variance model, and study-to-study heterogeneity was assessed using the I^2 test statistic in combination with visual inspection using Review Manager.[16] For disposition time and length of stay, we used mean differences in time (minutes) and their corresponding 95% confidence intervals (95% CI). Where studies reported the median time to disposition decision and length of stay, attempts were made to contact the original authors for mean times and standard deviations. In the case where they were not available, we estimated them using an approach suggested by Wan et al.[17] which approximates reported medians and quartiles/ranges to corresponding mean and standard deviations robustly by also taking into account studies' sample sizes to avoid small study bias.

Results

Description of included studies

The combined total of the original search and update was 26,124 studies as summarized in PRISMA[18] diagram below (**figure 1**). 225 papers were included in the overall review, from these, nine studies relevant to POCT blood based panel tests were selected and reported here, including eight RCTs and one before-after study. Seven studies reported on general panel tests and two studies focused on cardiac panels.

In total, 19,562 participants were included (see study characteristics, **table 1**). The majority of participants were either adults or defined as being aged over 15 years, with the exception of one study[19] which recruited from a paediatric ED where all participants were aged under 21 years. All the studies were based in ED departments except one study[20] that was based in the Canadian ambulance service. Notably, there were no studies based in primary care that focused on panel testing for diagnosis in the acute setting, there were only studies that monitored patients with chronic disease or analysed single tests.

A variety of different POCT panel devices were used in the studies. Although there was variability regarding the specific tests performed by different devices, general panels always included basic metabolic parameters such as sodium, potassium and glucose. Creatinine and basic blood gas analysis such as total carbon dioxide and base excess were also commonly featured. Cardiac panels always included troponin in combination with BNP[20] or creatine kinase (myocardial type) and myoglobin.[21]

There was variation in participant inclusion criteria. Two studies included a representative sample of adult ED patients who needed blood tests,[22,23] and one included patients "whose physicians ordered a comprehensive metabolic panel."[24] Two studies randomized all patients seen in ED but limited inclusion to the trial to only those patients whose blood work fell entirely within capabilities of the POC devices used.[19,25] Only one study[26] recorded data on the number of patients who also required tests that were beyond the scope of the POCT device. Personal communication to authors was attempted to obtain this data from the other studies but it had either not been recorded or authors did not respond. Two studies

For peer review only - http://bmjopen.bmj.com/site/about/guidelines.xhtml

excluded patients who required critical care.[19,24] The cardiac panel studies were more specific in their inclusion criteria, including only patients with chest pain and/or dyspnoea[20] and also had more extensive exclusion criteria such as patients with myocardial infarction on ECG.[21]

Figure 2, summarises the key features of methodological assessment for the 8 included RCTs. In general, for the included RCTs methodological quality was variable, with the exception of one study[26] being at high risk or unclear for most domains. The before-after study[27] also assessed as 'high or 'unclear' on all domains; it was also 'high risk' for the confounders assessment as neither the baseline characteristics of participants nor the care pathway for the control group were described in detail.

Figure 1: PRISMA

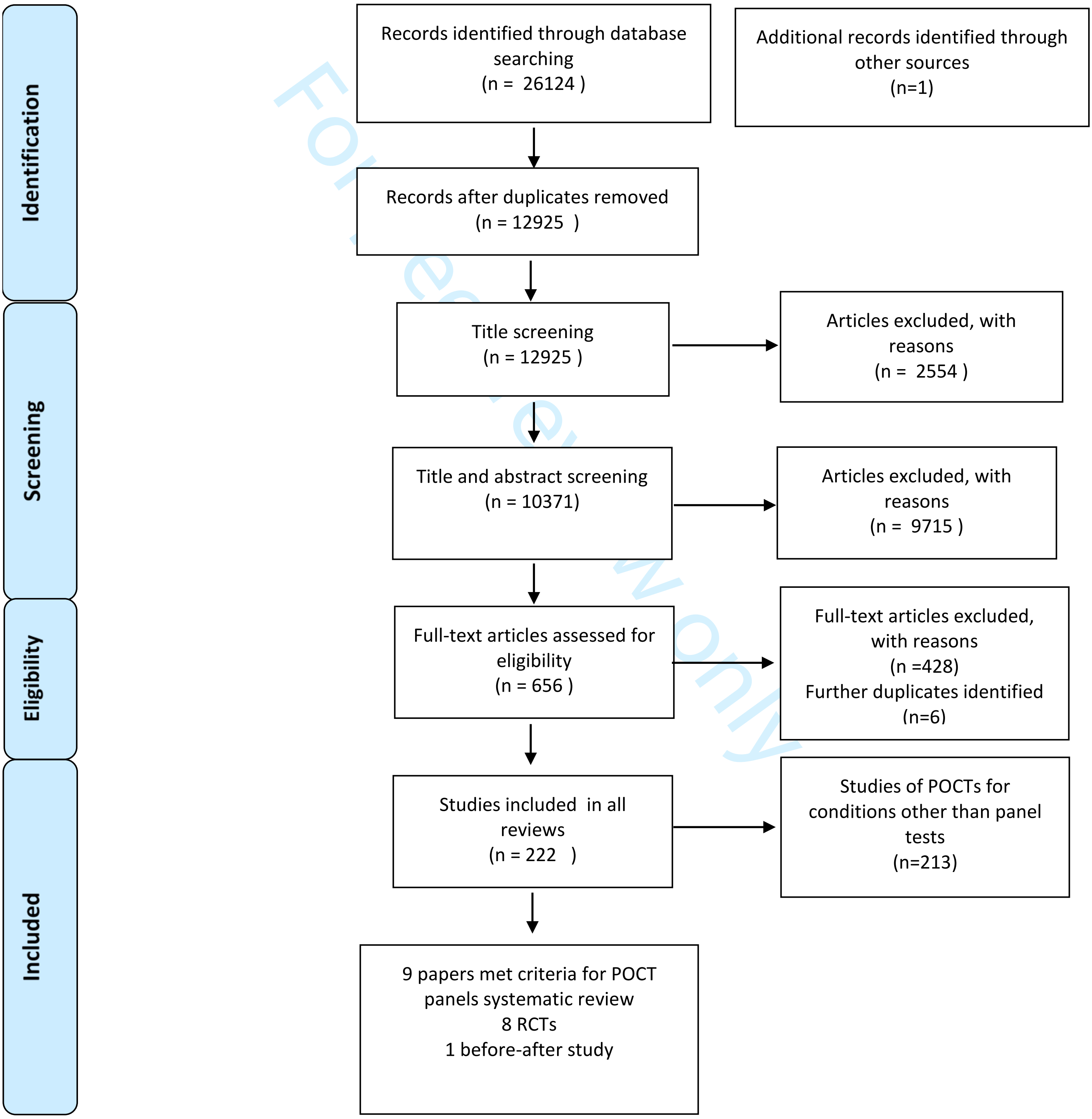

For peer review only - http://bmjopen.bmj.com/site/about/guidelines.xhtml

Table 1: Characteristics of included studies

*All studies were RCTs except from Parvin 1996(26) which was a before-after study design ** Assessed cardiac panels

Study	Setting	Device (Manufacturer)	Tests measured	Participant characteristics	Sample size
**Parvin* 1996**(27)	ED USA	i-STAT (i-STAT Corp, Princeton, NJ)	Sodium, potassium, chloride, blood urea nitrogen, glucose, haematocrit, haemoglobin	Patients presenting to the ED between Dec 1994-Jan 1995 (control period 1), Feb-April 1995 (intervention period) and April 1995 (control period 2) and who had blood tests done that were available on the i-STAT. Only 5.3% of patients had no other central laboratory testing performed in addition to i-STAT	2067
**Kendall 1998**(28)	ED<br>UK	i-STAT (Abbott)<br>2 cartridges evaluated	Sodium, potassium, chloride, urea, glucose, packed cell volume (PCV), calculates Hb from PCV<br>pH, partial pressure carbon dioxide (ppCO2), partial pressure oxygen (PPO2), bicarbonate, total CO2, base excess, oxygen saturation	Representative sample of adult ED patients who needed blood tests.<br>No exclusion criteria.	1728
**Murray 1998**(25)	ED<br>Canada	NOVA 16 CRT™ Spectral™ Cardiac STATus Test Kit	Creatinine, sodium, potassium, chloride, total CO2, glucose, blood urea nitrogen, haematocrit, qualitative CK-MB (creatine kinase MB isoenzyme) and myoglobin	Adult ED patients, all patients seen in ED randomized but only those patients **whose blood work fell entirely within capabilities of POC tests** were selected.	180
**Hsiao 2007**(19)	Tertiary paediatric ED<br>USA	i-STAT (Abbott)	Sodium, potassium, chloride, bicarbonate, glucose, blood urea nitrogen, creatinine, ionized calcium, haematocrit, basic blood gas analysis	Paediatric ED patients aged under 21 years old **who required blood work (solely) that the POC device was capable of performing.** Critically ill patients excluded.	239
**Lee 2011**(23)	Multicentre: 5 EDs<br>South Korea	Piccolo xpress device Piccolo Comprehensive Metabolic Reagent Discs	Protein, albumin, alk phos, alanine aminotransferase, aspartate aminotransferase, nitrogen, calcium, cr, glucose, postasium, sodium, bilirubin, total CO2	ED patients aged 15 years and older clinically required to have chemistry laboratory tests.	2323
**Illahi 2012**(26)	ED<br>UK	Siemens Dimension Xpand Plus analyser Sysmex XS 1000 analyser	Albumin, Alkaline phosphatase, Amylase, Bilirubin, Calcium, Creatinine, CRP, glucose, paracetamol, phosphate, potassium, sodium, urea, FBC, WBC and differential	Adult ED patients. Some samples then underwent further testing in the central laboratory if required tests were not available from POC device.	47
**Jang 2013**(24)	ED<br>Korea	Piccolo Xpress Chem Analyzer<br>Piccolo Comprehensive Metabolic Reagent Discs	Total protein, albumin, alkaline phosphatase, alanine aminotransferase, aspartate aminotransferase, urea, nitrogen, calcium, chloride, creatinine, glucose, potassium, sodium, total bilirubin and total carbon dioxide	ED patients aged 15 years and older whose physicians ordered a comprehensive metabolic panel.<br>Critically ill patients excluded.	10244
**Goodacre** 2011**(21)	Multicentre: 6 EDs UK	Siemens Stratus CS analyser cardiac panel	CK, myocardial type, myoglobin, troponin 1	Adult ED patients with chest pain. Several exclusion criteria applied including patients with ECG changes consistent with myocardial infarction/high risk acute coronary syndrome, confirmed or suspected serious non-coronary pathology.	2243
**Ezekowitz** 2014**(20)	Ambulance Service<br>Canada<br>(Out of hospital)	Alere Cardio2 panel	Troponin and B-type natriuretic peptide (BNP)	Adults > 18 years of age who activated emergency medical services (EMS) for acute chest discomfort or dyspnea for which acute cardiovascular disease was deemed to be the most probable diagnosis. Patients excluded if ST-elevation on ECG and non-cardiovascular cause suspected / recurrent dyspnea.	491

For peer review only - http://bmjopen.bmj.com/site/about/guidelines.xhtml

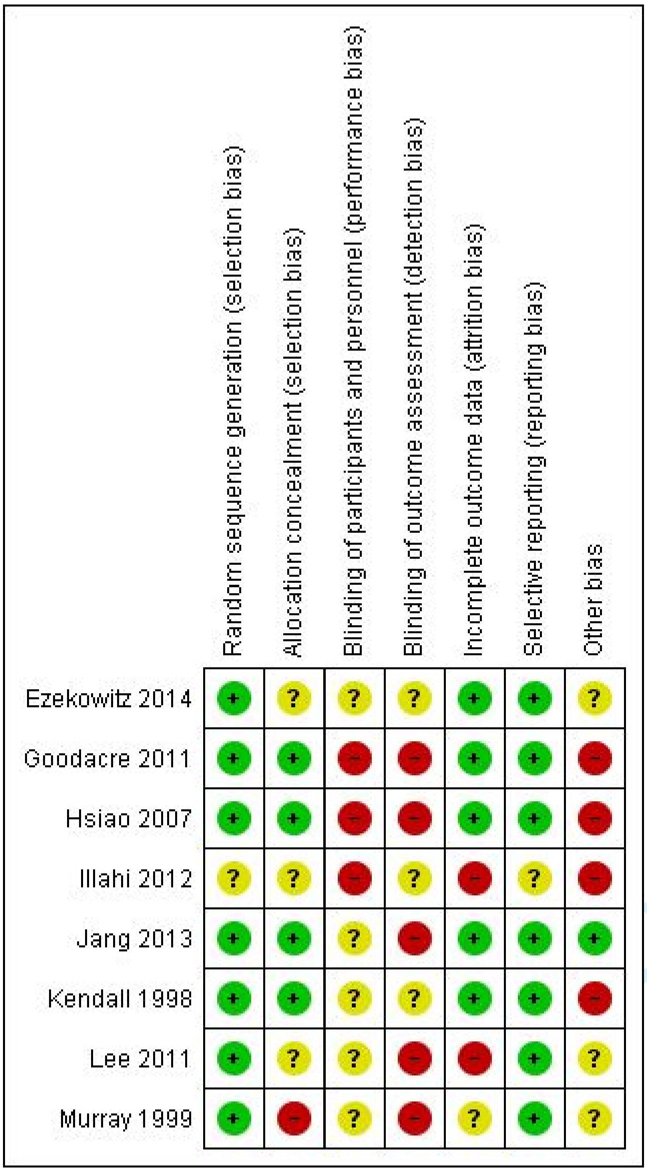

Figure 2: Risk of Bias Summary RCTs

Primary Outcome

Disposition Decision Time

The disposition decision (DD) time, was specifically reported in 3 studies.[14,19,26] As summarized in **figure 4**, POCT reduced the overall DD time by 40 minutes (95% CI -42.53 to -37.02, I^2=0%) compared to usual care. This reduction was increased to 48 minutes in patients who did not require additional laboratory tests (95% CI -61.16 to -34.10, I^2 =0%). Hsiao et al[19] recruited only from paediatric ED (patients aged under 21 years) whilst the other studies included adult patients. These all evaluated blood based panel POCT devices in general ED patients, but Hsiao only reports results for patients for whom only tests that fell within the capabilities of the POCT device were needed and Illahi et al[26] report these different subgroups of patients separately. Illahi et al[26] reported point estimates as average values and this has been taken as median values in our analysis, attempts were made to contact the author for confirmation but this was not successful. Sensitivity analysis excluding Illahi[26], demonstrated robust findings (Appendix A). Kendall et al[22] did not specifically measure DD so their results were not included in the meta-analysis, although they did describe that decisions regarding the management plan were made 74 minutes earlier (95% CI 68 min to 80 min, p<0.0001) when POCT was used for haematological tests as compared to central laboratory testing and 86 minutes earlier (80 min to 92 min p<0.0001) for biochemical tests.

For peer review only - http://bmjopen.bmj.com/site/about/guidelines.xhtml

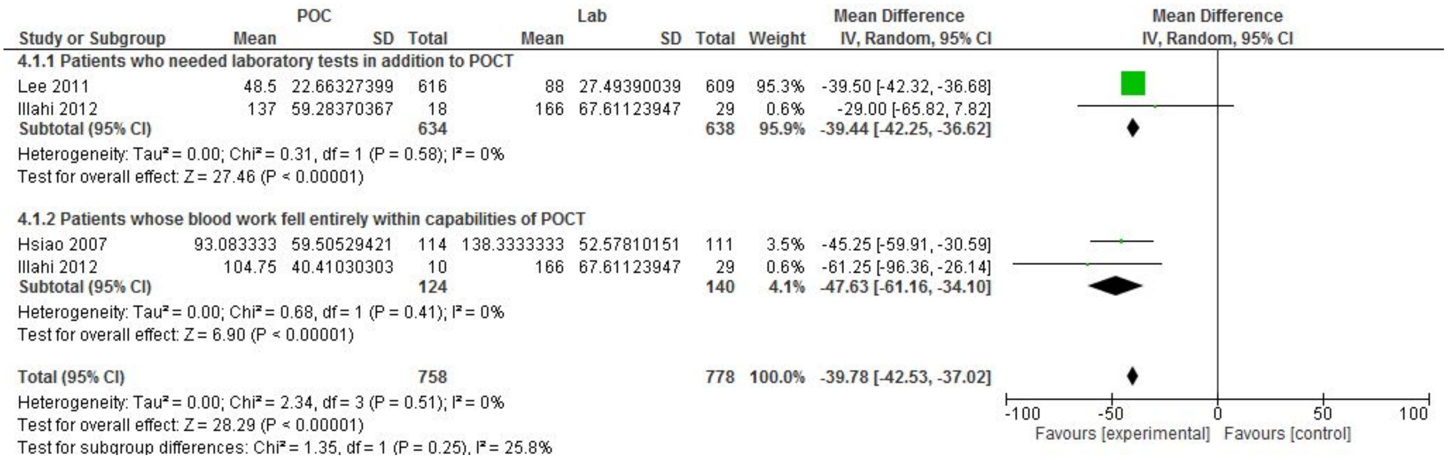

Figure 3: Time to Disposition Decision

Secondary outcomes

Length of Stay

LOS in ED was measured in six studies.[19,21,22,24,25,27] Four of these studies[19,22,24,25] were RCTs that assessed general POCT panel tests in ED and these were combined in the meta-analysis. These included three studies with adult participants[22,24,25] and one study[19] based in paediatric ED. A significant reduction in ED LOS of 33 minutes (95% CI -60.66 to -5.85) was observed in the POCT group although wide 95% confidence intervals were noted (**figure 5**). This reduction was increased to 37 minutes (95% CI -53.12 to -21.81) in patients who only required POCT (and needed no additional laboratory tests). Three of these studies[19,24,25] provided further specific data on the LOS for patients who were admitted and discharged. When this data was combined, POCT was found to reduce the overall LOS for patients who were later discharged by 34 minutes (95% CI -63.66 to -5.23) although wide CI were noted. There was no statistically significant difference between LOS in POCT versus usual care in patients who were later admitted (**figure 6**). In their before-after study of 4985 patients Parvin et al[27] evaluated a general POCT panel in ED; median LOS with POCT was 209 minutes (95% CI 111 to 368) versus 201 (95% CI 106-345) for usual care which was not statistically significant. Subgroup analysis by presenting symptoms and discharge/admit status did not detect any further differences.

LOS in ED was also measured in two studies on POCT cardiac panels.[20,21] One study integrated POCT into emergency medical services in Canada[20] and assessed patients with chest pain or dyspnoea, they found no difference in time from first medical contact to final disposition (9.2 (7.3-11.1) hours for the POCT group and 8.8 (6.3-12.1) hours for usual care (P=0.609). Goodacre et al[21] recorded successful discharge home from ED for patients with chest pain which they defined as having left hospital (or awaiting transport) within 4 hours of arrival and no adverse events occurring over the next 3 months. POCT cardiac biomarker panels were associated with an increased rate of successful discharge (32% vs 13% in the usual care group, OR 3.81, 95% CI 3.01-4.82; p<0.001), although analysis of the original data demonstrated that the median LOS in ED for the POCT group was longer at 216 minutes (IQR 179-238) compared to the usual care pathway of 188 minutes (IQR 142-225).

For peer review only

For peer review only - http://bmjopen.bmj.com/site/about/guidelines.xhtml

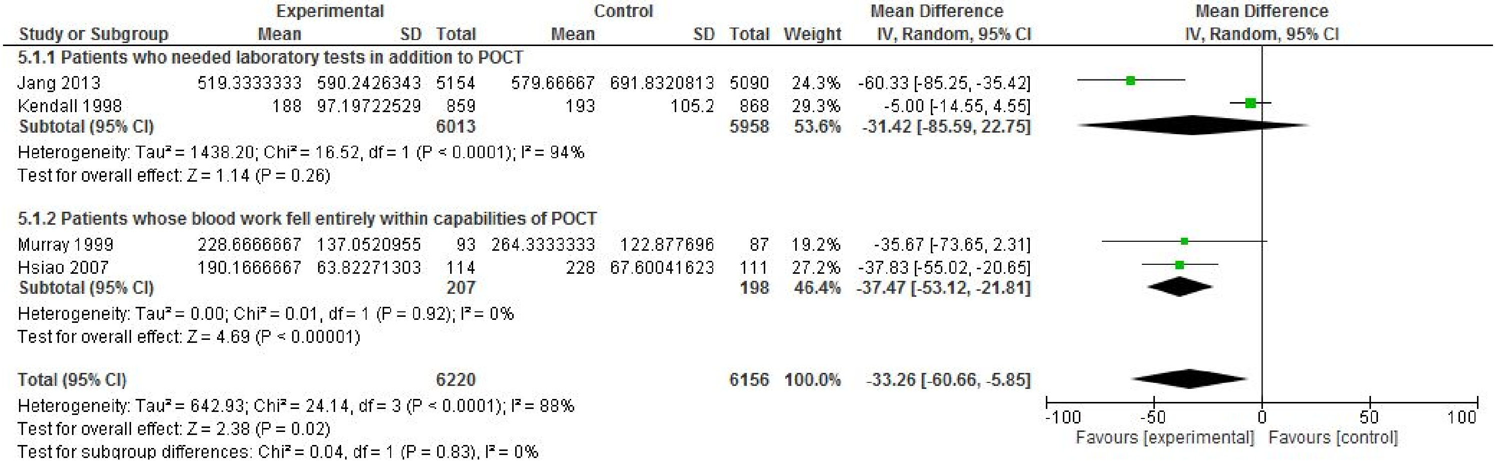

Figure 4: ED Length of stay

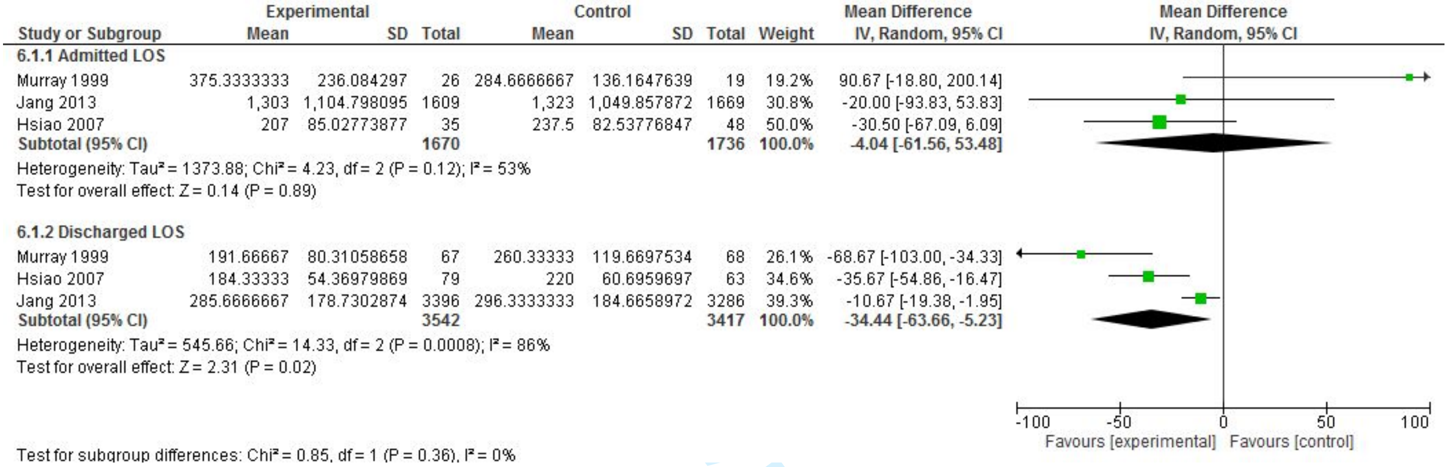

Figure 5: ED Length of stay (subgroups for admitted/discharged patients)

For the other secondary outcomes, only one study reported hospital admission rates[28] and found that this was not significantly different between the POCT and laboratory groups (difference 1.7, CI -1.7,5.1 P=0.33). Rates of repeat ED attendance after discharge and re-hospitalization were also recorded by Ezekowitz et al[20] and there was no significant difference detected between POCT and laboratory testing (P=0.320, P=0.712, respectively).

In terms of exploratory outcomes, there is evidence that unwell patients benefited from faster decision making with POCT. Kendall et al[22] describe how 59 out of 859 POCT patients had changes in their management in which timing was considered to be critical; these included decision to intubate/ventilate. POCT was also associated with reduced time to CT from ED arrival,[24] with a median difference of 11 minutes (95% CI 3 to 19).

Mortality

Three studies included data on patient mortality.[20,21,22] There was no significant difference in mortality between POCT and laboratory testing as demonstrated in **figure 6**. Two of these studies evaluated cardiac panels, calculated risk ratios of death were 2.98 (0.60,14.74)[21] and 0.80 (0.22, 2.94),[20] one study on general panels reported a relative risk of death of 1.16 (0.79,1.68).[22]

For peer review only - http://bmjopen.bmj.com/site/about/guidelines.xhtml

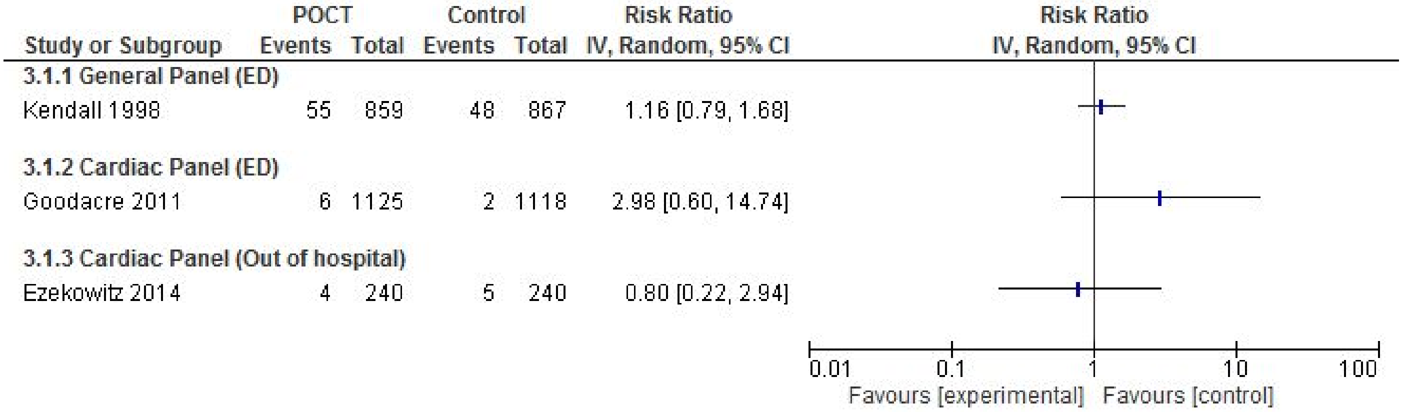

Figure 6: Relative risk of death in POCT versus laboratory groups

Discussion

Statement of principal findings

This systematic review found that general panel tests performed at the point-of-care may result in faster disposition and management decisions, which in turn might reduce LOS for patients who are subsequently discharged from the ED. This is not associated with changes in mortality. There is also no gain in LOS for patients who are admitted to hospital. These results perhaps suggest that specific groups of patients may benefit from the introduction of POCT in an ED setting; such as, well patients who could be discharged faster and unwell patients who need critical interventions more quickly. The LOS advantage was attenuated when extra tests were required from the laboratory in addition to the POCT panel.

Strengths and weaknesses of the study

This study was conducted robustly, using a comprehensive search strategy in the major medical databases, and selection and quality assessment performed by two independent reviewers. It offers results on the impact of panel point-of-care tests, rather than just their accuracy. Although impact studies are an integral part of the evidence cycle for new tests,[28] they are also difficult to organize and subject to bias. In our review, blinding clinicians and patients from the intervention was not possible by nature, introducing a risk of bias and in general most studies were not blinded by outcome assessment. Studies were also relatively small, and most notably for mortality, the results may suffer from power problems.

Statistical and clinical heterogeneity is evident within our meta-analysis, particularly for LOS results. The meta-analysis was considered carefully and reduced where possible by ensuring that studies of cardiac and general panels tests were not combined; moreover, we did not combine the before-after study (which also had a high risk of bias)[27] with RCTs. Multiple factors influence our primary and secondary outcomes and these variables are responsible for much of the clinical heterogeneity. It is important to consider the system in which POCT is implemented and which ED triage systems are used. For example, if blood tests are requested on arrival in ED than laboratory results might be available at the time of physician review anyway and thus there would be fewer benefits to POCT. Moreover, practicalities such as how quickly radiology is available and how samples are transported to laboratories will impact results significantly. There are also many factors which impact ED LOS specifically, especially availability of inpatient beds and this may be the reason for the reduced benefit on LOS in the admitted group. Other important factors that differed between the studies and between different hospitals,[21] included the time of day that POCT was available; with one study only performing POCT during working hours, as well as the availability and

For peer review only

For peer review only - http://bmjopen.bmj.com/site/about/guidelines.xhtml

seniority of clinical staff. Furthermore, the studies differed in their inclusion criteria, and as demonstrated by our subgroup analysis, the benefits of POCT on LOS was proportional to the spectrum of tests available. This perhaps explains why Parvin et al[27] did not demonstrate any benefit in reduced LOS from POCT as 95% of these patients also required additional laboratory tests in addition to the POCT panel.[28] Moreover, there is further evidence of this association from other studies that combined single and multiple tests and demonstrated a significant reduction in LOS for POCT.[29,30]

Other factors relate specifically to study protocol, for example Goodacre et al[21] describe how the LOS was longer for the POCT because POCT patients did not leave the ED until their POCT testing (at baseline and 90 minutes) was complete, whereas the standard care group could leave the ED as soon as medical assessment was complete and a decision to admit (or discharge in a few cases) had been made. Therefore, the POCT group spent longer in the ED but were more likely to go home before the 4 hour point, whilst the usual care group spent less time in the ED because they were more likely to be admitted to a ward (and thus leave the ED) at any earlier time (personal communication with author).

An important limitation to highlight is that four studies excluded critically ill patients [19,24] and patients with myocardial infarction,[20,21] which may have biased mortality data.

Comparison with other studies

The benefit of POCT in ambulatory patients has been shown by Kankaanpaa et al,[31] where single and panel tests were implemented in ambulatory patients presenting to a Finnish ED who also see primary care patients outside of office hours. They excluded all patients who were admitted to hospital. Median LOS in the control phase was 3.51 hours (3.38-4.04) and this was reduced to 3.22 hours (3.12-3.31 p=0.000) with the implementation of POCT; moreover, the combination of POCT with an early assessment triage model, reduced LOS to 3.05 hours (02.59-03.12, p=0.033). This study[31] appropriately recorded which patients also required additional laboratory testing and found that this was lowest when POCT and an early assessment triage model were combined, when 68% of patients did not require additional blood tests (which was also associated with the greatest reduction in LOS).

Implications for research and practice

Future research is required to understand the impact that POCT panels have in assisting with the decision to admit or discharge patients and analyse their cost-effectiveness.[32,33] We would recommend that future trials assess successful discharge, rate of admission and rate of adverse events rather than just focussing on time to discharge or disposition decision. Moreover, the relationship between ED overcrowding and LOS needs to be better understood as reductions in LOS do not necessarily reduce overcrowding.[34] This review suggests that there are specific subgroups that may benefit most from the implementation of POCT and future studies should focus on these groups and establish which tests should be combined in a POCT panel such as CRP.

Theoretically there are also advantages to using POCT in the primary care setting. For example, it may help to identify acute kidney injury, venous thromboembolism or atypical presentation of myocardial infarction. However, it may not be time efficient or cost effective. As none of the included studies were based in primary care, understanding the impact of POCT in this setting remains a research priority. Research outcomes and study designs in this environment need to be carefully considered, particularly as laboratory testing may not be available at all or maybe delayed, particularly regarding home visits and for patients in rural areas. It is important to understand how POCT changes management decisions particularly regarding admission and to monitor whether the thresholds for ordering tests changes with POCT implementation.[34]

For peer review only - http://bmjopen.bmj.com/site/about/guidelines.xhtml

Any future research should identify how POCT can contribute meaningful changes to patient care rather than simply look at health care processes and should also consider the patients perspective.

What this paper adds:

> The technology behind point-of-care testing has developed extensively and their implementation in day to day practice is expected to increase substantially. However, little is understood about the impact of point of care testing on patients and health care processes.
>
> This systematic review and meta-analysis found that general panel tests performed at the point-of-care in ED and pre-hospital care settings, may result in faster disposition and management decisions, which in turn might reduce LOS for patients who are subsequently discharged from the ED.

Copyright: I, Clare R Goyder, the Submitting Author has the right to grant and does grant on behalf of all authors of the Work (as defined in the below author licence), an exclusive licence and/or a non-exclusive licence for contributions from authors who are: i) UK Crown employees; ii) where BMJ has agreed a CC BY licence shall apply, and/or iii) in accordance with the terms applicable for US Federal Government officers or employees acting as part of their official duties; on a worldwide, perpetual, irrevocable, royalty-free basis to BMJ Publishing Group Ltd ("BMJ") its licensees and where the relevant Journal is co-owned by BMJ to the co-owners of the Journal, to publish the Work in EMJ and any other BMJ products and to exploit all rights, as set out in our licence.

Contributors: TA designed the initial search strategy. Screening was carried out by JV, JL, CG, PST, TA, PT, AV. Data extraction was performed by CG, AV and TA. CG and PST carried out the meta-analysis. CG and AV drafted the manuscript. All authors commented and co-drafted the final version of this article. CG is the guarantor and affirms that the manuscript is an honest, accurate, and transparent account of the study being reported; that no important aspects of the study have been omitted; and that any discrepancies from the study as planned (and, if relevant, registered) have been explained.

Funding: This paper presents independent research funded by the National Institute for Health Research School for Primary Care Research (NIHR SPCR), funding round 11, award number 309. CG is a Wellcome Trust Doctoral Fellow. JV, GH and AV are supported through the NIHR Community Healthcare MedTech and IVD Co-operative. JL is an NIHR In Practice Fellow.

Disclaimer: The views expressed are those of the author(s) and not necessarily those of the NIHR, the NHS or the Department of Health

Competing interests: All authors have completed the Unified Competing Interest form (available on request from the corresponding author) and declare: no support from any organisation for the submitted work [or describe if any]; no financial relationships with any organisations that might have an interest in the submitted work in the previous three years [or describe if any], no other relationships or activities that could appear to have influenced the submitted work [or describe if any].

Ethical approval: Not required.

Systematic review registration PROSPERO CRD42016035426

Acknowledgements: We thank Nia Roberts, Dr Jennifer Hirst and Dr José M. Ordóñez-Mena for their

assistance with this project.

References

1. Kostopoulou O, Delaney BC, Munro CW. Diagnostic difficulty and error in primary care - A systematic review. Vol. 25, Family Practice. 2008. p. 400–13.
2. Verbakel JY, Aertgeerts B, Lemiengre M, Sutter A De, Bullens DMA, Buntinx F. Analytical accuracy and user-friendliness of the Afinion point-of-care CRP test. J Clin Pathol [Internet]. 2014;67(1):83–6. Available from: http://jcp.bmj.com/lookup/doi/10.1136/jclinpath-2013-201654
3. Luukkonen AAM, Lehto TM, Hedberg PSM, Vaskivuo TE. Evaluation of a hand-held blood gas analyzer for rapid determination of blood gases, electrolytes and metabolites in intensive care setting. Clin Chem Lab Med. 2016;54(4):585–94.
4. Abel G. Expert Review of Molecular Diagnostics Current status and future prospects of point-of-care testing around the globe. Expert Rev Mol Diagn [Internet]. 2015;15(7):853–5. Available from: http://www.tandfonline.com/action/journalInformation?journalCode=iero20
5. Sohn AJ, Hickner JM, Alem F. Use of Point-of-Care Tests (POCTs) by US Primary Care Physicians. J Am Board Fam Med [Internet]. 2016;29(3):371–6. Available from: http://www.jabfm.org/cgi/doi/10.3122/jabfm.2016.03.150249
6. NHS England Urgent Treatment Centres [Internet]. 2017. Available from: https://www.england.nhs.uk/wp-content/uploads/2017/07/urgent-treatment-centres–principles-standards.pdf [Accessed 7th June 2018]
7. Cals JWL, Schols AMR, van Weert HCPM, Stevens F, Zeijen CGIP, Holtman G, et al. [Point-of-care testing in family practices: present use and need for tests in the future]. Ned Tijdschr Geneeskd [Internet]. 2014;158:A8210. Available from: http://www.ncbi.nlm.nih.gov/pubmed/25515388
8. Turner PJ, Van den Bruel A, Jones CHD, Plüddemann A, Heneghan C, Thompson MJ, et al. Point-of-care testing in UK primary care: A survey to establish clinical needs. Fam Pract. 2016;33(4):388–94.
9. Howick J, Cals JWL, Jones C, Price CP, Pluddemann A, Heneghan C, et al. Current and future use of point-of-care tests in primary care: an international survey in Australia, Belgium, The Netherlands, the UK and the USA. BMJ Open [Internet]. 2014;4(8):e005611–e005611. Available from: http://bmjopen.bmj.com/cgi/doi/10.1136/bmjopen-2014-005611
10. Gutierres SL, Welty TE. Point-of-Care Testing: An Introduction. Vol. 38, Annals of Pharmacotherapy. 2004. p. 119–25.
11. Higgins JP, Green S. Cochrane Handbook for Systematic Reviews of [Internet]. Vol. Version 5., The Cochrane Collaboration. 2008. Version 5.1.0. Available from: http://onlinelibrary.wiley.com/doi/10.1002/9780470712184.fmatter/summary
12. Moher D, Liberati A, Tetzlaff J, Altman DG, The PRISMA Group (2009) Preferred Reporting Items for Systematic Reviews and Meta-Analyses: The PRISMA Statement. PLoS Med 6(7): e1000097. https://doi.org/10.1371/journal.pmed.1000097
13. Verbakel JY, Lee JJ, Goyder CR, Tan PS, Ananthakumar T, Turner PJ, l Hayward G, Van den Bruel A. The impact of point-of-care c-reactive protein in ambulatory care: A systematic review and meta-analysis. BMJ Open. 2019; 1:9 (1):e025036
14. Lee JJ, Verbakel JY, Goyder CR, Ananthakumar T, Tan PS, Turner PJ, Hayward G, Van den Bruel A. Clinical Infectious Diseases. 2018; ciy837, https://doi.org/10.1093/cid/ciy837
15. Reeves C B, Deeks J J, Higgins P.,T. J, Wells A G. Cochrane Handbook: Special Topics: Ch 13: Including non-randomized studies. In: Cochrane Handbook for: Systematic Reviews of Interventions [Internet]. 2011. p. 389–432. Available from: http://training.cochrane.org/handbook [Accessed 7th June 2018)
16. Review Manager (RevMan). No Title. Review Manager (RevMan) Version 5.3. Copenhagen: The Nordic Cochrane Centre, The Cochrane Collaboration, 2014.
17. Wan X, Wang W, Liu J, Tong T. Estimating the sample mean and standard deviation from the sample size, median, range and/or interquartile range. BMC Med Res Methodol. 2014;14(1).

For peer review only - http://bmjopen.bmj.com/site/about/guidelines.xhtml

18. Prisma Flow Diagram [Internet]. Available from: http://prisma-statement.org/prismastatement/flowdiagram.aspx [accessed 13th June 2018]
19. Hsiao AL, Santucci KA, Dziura J, Baker MD. A randomized trial to assess the efficacy of point-of-care testing in decreasing length of stay in a pediatric emergency department. Pediatr Emerg Care [Internet]. 2007;23(7):457–62. Available from: http://www.ncbi.nlm.nih.gov/pubmed/17666926
20. Ezekowitz JA, Welsh RC, Gubbels C, Brass N, Chan M, Keeble W, et al. Providing rapid out of hospital acute cardiovascular treatment 3 (PROACT-3). Can J Cardiol. 2014;30(10):1208–15.
21. Goodacre SW, Bradburn M, Cross E, Collinson P, Gray A, Hall AS. The Randomised Assessment of Treatment using Panel Assay of Cardiac Markers (RATPAC) trial: A randomised controlled trial of point-of-care cardiac markers in the emergency department. Heart. 2011;97(3):190–6.
22. Kendall J, Reeves B, Clancy M. Point of care testing: randomised controlled trial of clinical outcome. BMJ. 1998;316(7137):1052–7.
23. Lee EJ, Shin S Do, Song KJ, Kim SC, Cho JS, Lee SC, et al. A point-of-care chemistry test for reduction of turnaround and clinical decision time. In: American Journal of Emergency Medicine. 2011. p. 489–95.
24. Jang JY, Shin S Do, Lee EJ, Park CB, Song KJ, Singer AJ. Use of a comprehensive metabolic panel point-of-care test to reduce length of stay in the emergency department: A randomized controlled trial. Ann Emerg Med. 2013;61(2):145–51.
25. Murray RP, Leroux M, Sabga E, Palatnick W, Ludwig L. Effect of point of care testing on length of stay in an adult Emergency Department. J Emerg Med. 1999;17(5):811–4.
26. Illahi MN, Lapworth R, Bates P. The effect of point of care testing on clinical decision making in an emergency department [Internet]. Vol. 11, Journal of the Liaquat University of Medical and Health Sciences. 2012: 153–7.
27. Parvin CA, Lo SF, Deuser SM, Weaver LG, Lewis LM, Scott MG. Impact of point-of-care testing on patients' length of stay in a large emergency department. Clin Chem. 1996;42(5):711–7.
28. Verbakel JY, Turner PJ, Thompson MJ, Plüddemann A, Price CP, Shinkins B, et al. Common evidence gaps in point-of-care diagnostic test evaluation: A review of horizon scan reports. BMJ Open. 2017;7(9).
29. Lee-Lewandrowski E, Corboy D, Lewandrowski K, Sinclair J, McDermot S, Benzer TI. Implementation of a point-of-care satellite laboratory in the emergency department of an academic medical center: Impact on test turnaround time and patient emergency department length of stay. Arch Pathol Lab Med. 2003;127(4):456–60.
30. Asha SE, Chan ACF, Walter E, Kelly PJ, Morton RL, Ajami A, et al. Impact from point-of-care devices on emergency department patient processing times compared with central laboratory testing of blood samples: a randomised controlled trial and cost-effectiveness analysis. Emerg Med J [Internet]. 2014 Sep 1 [cited 2018 Mar 7];31(9):714–9. Available from: http://www.ncbi.nlm.nih.gov/pubmed/23748157
31. Kankaanpaa M, Raitakari M, Muukkonen L, Gustafsson S, Heitto M, Palomaki A, et al. Use of point-of-care testing and early assessment model reduces length of stay for ambulatory patients in an emergency department. Scand J Trauma Resusc Emerg Med [Internet]. 2016;24(1):125. Available from: http://dx.doi.org/10.1186/s13049-016-0319-z
32. Fermann GJ, Suyama J. Clinical Laboratory in Emergency Medicine Point of care testing in the emergency department Available from: https://ac.els-cdn.com/S0736467902004298/1-s2.0-S0736467902004298-main.pdf?_tid=63c3e414-0592-4cc5-a81b-37abed7224f9&acdnat=1520427388_2833d860886622e640aa71267a9157dd
33. Tsai WW, Nash DB, Seamonds B, Weir GJ. Point-of-care versus central laboratory testing: an economic analysis in an academic medical center. Clin Ther [Internet]. 1994 [cited 2018 Mar 26];16(5):898–910; discussion 854. Available from: http://www.ncbi.nlm.nih.gov/pubmed/7859247
34. Goodacre SW. The Practice Of Emergency Medicine/Editorial. Point-of-Care Testing May Reduce Length of Stay but Not Emergency Department Crowding. Ann Emerg Med [Internet]. 2012 [cited

For peer review only - http://bmjopen.bmj.com/site/about/guidelines.xhtml

2018 Mar 7];61:153–4. Available from: http://dx.doi.org/10.1016/j.annemergmed.2012.09.001

For peer review only

For peer review only - http://bmjopen.bmj.com/site/about/guidelines.xhtml

For peer review only

For peer review only - http://bmjopen.bmj.com/site/about/guidelines.xhtml

Appendix A

Sensitivity Analysis

Figure 4: Disposition Decision (subgroups for patients who needed laboratory tests in addition to POCT)

Figure 4a) With Illahi (25)

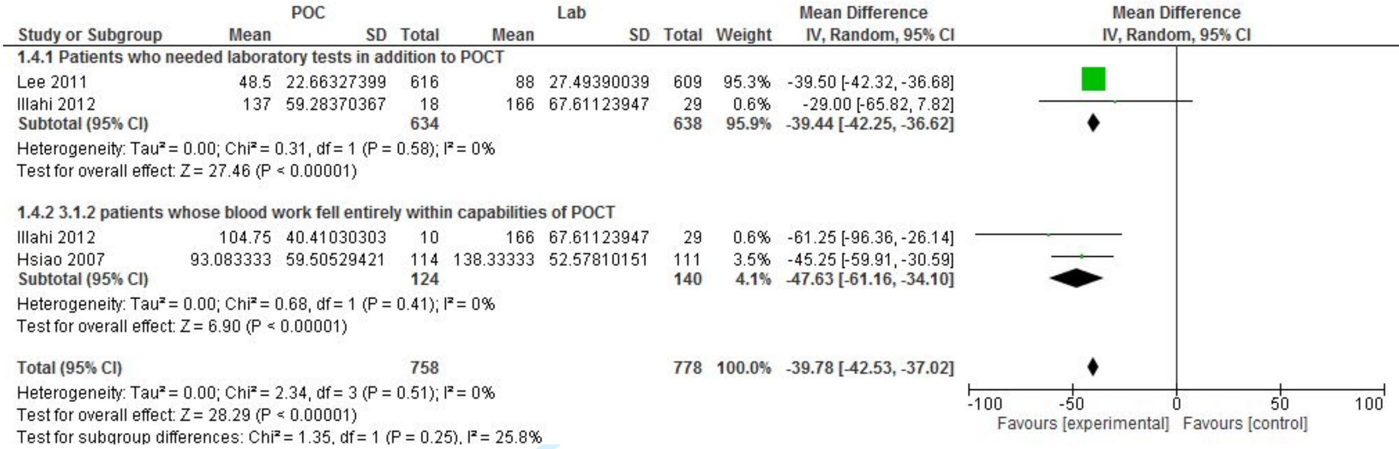

Figure 4b) Without Illahi (25)

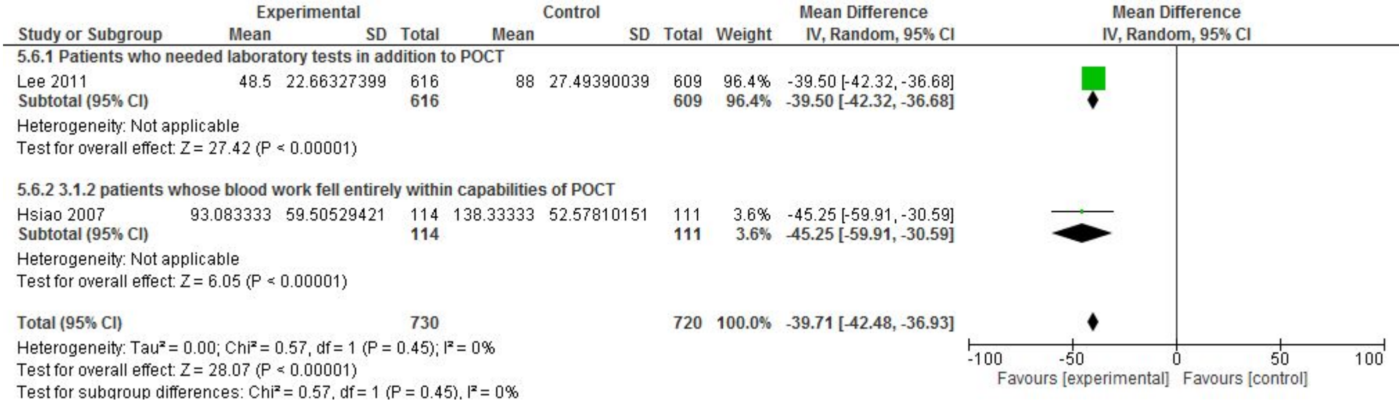

Appendix B : Search Strategy

1	Ambulatory Care/
2	exp Ambulatory Care Facilities/
3	general practice/ or family practice/
4	general practitioners/ or physicians, family/ or physicians, primary care/
5	Primary Health Care/
6	Office Visits/
7	exp Emergency Service, Hospital/
8	Emergency Medical Services/
9	(ambulatory adj3 (care or setting? or facilit* or ward? or department? or service?)).ti,ab.
10	((general or family) adj2 (practi* or physician? or doctor?)).ti,ab.
11	(primary care or primary health care or primary healthcare).ti,ab.
12	(emergency adj3 (care or setting? or facilit* or ward? or department? or service?)).ti,ab.
13	(after hour? or afterhour? or "out of hour?" or ooh).ti,ab.
14	(clinic? or visit?).ti,ab.
15	((health* or medical) adj2 (center? or centre?)).ti,ab.
16	community health services/ or exp community health nursing/
17	Community Health Workers/
18	(community adj2 (health or health care or service? or program*)).ti,ab.
19	(community adj2 (worker? or aide? or volunteer? or assistant? or visitor?)).ti,ab.
20	((lay or volunteer) adj2 (health worker? or health aide? or health assistant?)).ti,ab.
21	((health* or medical) adj2 (facility or facilities)).ti,ab.
22	1 or 2 or 3 or 4 or 5 or 6 or 7 or 8 or 9 or 10 or 11 or 12 or 13 or 14 or 15
23	16 or 17 or 18 or 19 or 20 or 21
24	Point-of-Care Systems/
25	(("point of care" or POC) adj3 (test* or diagnos*)).ti,ab.
26	(("point of care" or POC) and (test* or diagnos*)).ti.
27	poct.ti,ab.
28	((rapid or bedside or bed-side or "near patient") adj3 (test* or diagnos*)).ti,ab.
29	((rapid or bedside or bed-side or "near patient") and (test* or diagnos*)).ti.
30	24 or 25 or 26 or 27 or 28 or 29
31	(istat or i-stat or afinion).ti,ab.
32	30 or 31
33	22 and 32
34	23 and 32
35	34 not 33

For peer review only

PRISMA 2009 Checklist

Section/topic	#	Checklist item	Reported on page #
**TITLE**			
Title	1	Identify the report as a systematic review, meta-analysis, or both.	1
**ABSTRACT**			
Structured summary	2	Provide a structured summary including, as applicable: background; objectives; data sources; study eligibility criteria, participants, and interventions; study appraisal and synthesis methods; results; limitations; conclusions and implications of key findings; systematic review registration number.	2
**INTRODUCTION**			
Rationale	3	Describe the rationale for the review in the context of what is already known.	3
Objectives	4	Provide an explicit statement of questions being addressed with reference to participants, interventions, comparisons, outcomes, and study design (PICOS).	3
**METHODS**			
Protocol and registration	5	Indicate if a review protocol exists, if and where it can be accessed (e.g., Web address), and, if available, provide registration information including registration number.	3
Eligibility criteria	6	Specify study characteristics (e.g., PICOS, length of follow-up) and report characteristics (e.g., years considered, language, publication status) used as criteria for eligibility, giving rationale.	4
Information sources	7	Describe all information sources (e.g., databases with dates of coverage, contact with study authors to identify additional studies) in the search and date last searched.	4
Search	8	Present full electronic search strategy for at least one database, including any limits used, such that it could be repeated.	Appendix A
Study selection	9	State the process for selecting studies (i.e., screening, eligibility, included in systematic review, and, if applicable, included in the meta-analysis).	4
Data collection process	10	Describe method of data extraction from reports (e.g., piloted forms, independently, in duplicate) and any processes for obtaining and confirming data from investigators.	4
Data items	11	List and define all variables for which data were sought (e.g., PICOS, funding sources) and any assumptions and simplifications made.	4
Risk of bias in individual studies	12	Describe methods used for assessing risk of bias of individual studies (including specification of whether this was done at the study or outcome level), and how this information is to be used in any data synthesis.	4
Summary measures	13	State the principal summary measures (e.g., risk ratio, difference in means).	5
Synthesis of results	14	Describe the methods of handling data and combining results of studies, if done, including measures of consistency (e.g., I^2) for each meta-analysis.	5

For peer review only

PRISMA 2009 Checklist

Section/topic	#	Checklist item	Reported on page #
Risk of bias across studies	15	Specify any assessment of risk of bias that may affect the cumulative evidence (e.g., publication bias, selective reporting within studies).	4
Additional analyses	16	Describe methods of additional analyses (e.g., sensitivity or subgroup analyses, meta-regression), if done, indicating which were pre-specified.	4
**RESULTS**			
Study selection	17	Give numbers of studies screened, assessed for eligibility, and included in the review, with reasons for exclusions at each stage, ideally with a flow diagram.	6
Study characteristics	18	For each study, present characteristics for which data were extracted (e.g., study size, PICOS, follow-up period) and provide the citations.	7
Risk of bias within studies	19	Present data on risk of bias of each study and, if available, any outcome level assessment (see item 12).	8
Results of individual studies	20	For all outcomes considered (benefits or harms), present, for each study: (a) simple summary data for each intervention group (b) effect estimates and confidence intervals, ideally with a forest plot.	9-12
Synthesis of results	21	Present results of each meta-analysis done, including confidence intervals and measures of consistency.	9-12
Risk of bias across studies	22	Present results of any assessment of risk of bias across studies (see Item 15).	8
Additional analysis	23	Give results of additional analyses, if done (e.g., sensitivity or subgroup analyses, meta-regression [see Item 16]).	Appendix A
**DISCUSSION**			
Summary of evidence	24	Summarize the main findings including the strength of evidence for each main outcome; consider their relevance to key groups (e.g., healthcare providers, users, and policy makers).	11
Limitations	25	Discuss limitations at study and outcome level (e.g., risk of bias), and at review-level (e.g., incomplete retrieval of identified research, reporting bias).	11-12
Conclusions	26	Provide a general interpretation of the results in the context of other evidence, and implications for future research.	11-13
**FUNDING**			
Funding	27	Describe sources of funding for the systematic review and other support (e.g., supply of data); role of funders for the systematic review.	13

From: Moher D, Liberati A, Tetzlaff J, Altman DG, The PRISMA Group (2009). Preferred Reporting Items for Systematic Reviews and Meta-Analyses: The PRISMA Statement. PLoS Med 6(7): e1000097. doi:10.1371/journal.pmed1000097

For more information, visit: **www.prisma-statement.org**.

For peer review only - http://bmjopen.bmj.com/site/about/guidelines.xhtml

Impact of point-of-care panel tests in ambulatory care: a systematic review and meta-analysis

Journal:	*BMJ Open*
Manuscript ID	bmjopen-2019-032132.R1
Article Type:	Original research
Date Submitted by the Author:	22-Nov-2019
Complete List of Authors:	Goyder, Clare; University of Oxford, Nuffield Department of Primary Care Health Sciences<br>Tan, Pui San; University of Oxford, Nuffield Department of Primary Care Health Sciences<br>Verbakel, Jan; University of Oxford, Nuffield Department of Primary Care Health Sciences; KU Leuven, Department of Public Health and Primary Care<br>Ananthakumar, Thanusha; University of Oxford, Nuffield Department of Primary Care Health Sciences<br>Lee, Joseph; University of Oxford, Nuffield Department of Primary Care Health Sciences<br>Hayward, Gail; University of Oxford, Nuffield Department of Primary Care Health Sciences<br>Turner, Philip; University of Oxford, Nuffield Department of Primary Care Health Sciences<br>Van Den Bruel, Ann; KU Leuven, Department of Public Health and Primary Care
**Primary Subject Heading**:	Emergency medicine
Secondary Subject Heading:	General practice / Family practice, Emergency medicine, Evidence based practice
Keywords:	point of care tests, POCT, ACCIDENT & EMERGENCY MEDICINE, PRIMARY CARE, Paediatric A&E and ambulatory care < PAEDIATRICS, CHEMICAL PATHOLOGY

SCHOLARONE™
Manuscripts

I, the Submitting Author has the right to grant and does grant on behalf of all authors of the Work (as defined in the below author licence), an exclusive licence and/or a non-exclusive licence for contributions from authors who are: i) UK Crown employees; ii) where BMJ has agreed a CC-BY licence shall apply, and/or iii) in accordance with the terms applicable for US Federal Government officers or employees acting as part of their official duties; on a worldwide, perpetual, irrevocable, royalty-free basis to BMJ Publishing Group Ltd ("BMJ") its licensees and where the relevant Journal is co-owned by BMJ to the co-owners of the Journal, to publish the Work in this journal and any other BMJ products and to exploit all rights, as set out in our licence.

The Submitting Author accepts and understands that any supply made under these terms is made by BMJ to the Submitting Author unless you are acting as an employee on behalf of your employer or a postgraduate student of an affiliated institution which is paying any applicable article publishing charge ("APC") for Open Access articles. Where the Submitting Author wishes to make the Work available on an Open Access basis (and intends to pay the relevant APC), the terms of reuse of such Open Access shall be governed by a Creative Commons licence – details of these licences and which Creative Commons licence will apply to this Work are set out in our licence referred to above.

Other than as permitted in any relevant BMJ Author's Self Archiving Policies, I confirm this Work has not been accepted for publication elsewhere, is not being considered for publication elsewhere and does not duplicate material already published. I confirm all authors consent to publication of this Work and authorise the granting of this licence.

For peer review only

Impact of point-of-care panel tests in ambulatory care: a systematic review and meta-analysis

Clare R Goyder, GP/Clinical Researcher[1]
Pui San Tan, Researcher[1]
Jan Y Verbakel, GP/Assistant professor[1, 2]
Thanusha Ananthakumar, GP/Clinical Researcher[1]
Joseph Lee, GP/Clinical Researcher[1]
Gail Hayward, GP/Academic Clinical Lecturer[1]
Philip Turner, Researcher[1]
Ann Van den Bruel, GP/Associate professor[2]

1. Nuffield Department of Primary Care Health Sciences, University of Oxford, Oxford OX2 6GG, UK

2. Department of Public Health and Primary Care, KU Leuven
   Kapucijnenvoer 33, 3000 Leuven, Belgium

Corresponding author: clare.goyder@phc.ox.ac.uk

For peer review only - http://bmjopen.bmj.com/site/about/guidelines.xhtml

Abstract

Objectives This article summarizes all the available evidence on the impact of introducing blood-based point-of-care panel testing in ambulatory care on patient outcomes and healthcare processes.

Design Systematic review and meta-analysis of randomised controlled trials and before-after studies.

Data Sources Ovid Medline, Embase, Cochrane Database of Systematic Reviews, Cochrane CENTRAL, Database of Abstracts of Reviews and Effects, Science Citation Index from inception to 22nd October 2019.

Eligibility criteria for selecting studies Included studies were based in ambulatory care and compared point-of-care panel tests with laboratory testing. The primary outcome was the time to decision regarding disposition i.e. admission/referral termed disposition decision time. Secondary outcomes included length of stay at the ambulatory care unit/practice and mortality.

Results 19,562 patients from nine studies were included in the review, eight of these were RCTs, and one was a before-after study. All the studies were based in either emergency departments or the ambulance service; no studies were from primary care settings. General panel tests performed at the point-of-care resulted in disposition decisions being made 40 minutes faster (95% CI -42.2 to -36.6, I^2=0%) compared to the group receiving usual care, including central laboratory testing. This in turn resulted in a reduction in length of stay for patients who were subsequently discharged by 34 minutes (95% CI -63.7 to -5.16). No significant difference in mortality was reported.

Discussion Although statistical and clinical heterogeneity is evident and only a small number of studies were included in the meta-analysis, our results suggest that point-of-care panel tests might lead to faster discharge decisions. Future research should be performed in primary care and identify how point-of-care tests can contribute meaningful changes to patient care rather than focussing on healthcare processes.

Systematic review registration PROSPERO CRD42016035426

For peer review only

Strengths and limitations of this study

- This study was conducted robustly, following Preferred Reporting Items for Systematic Reviews and Meta-analyses guidelines using a comprehensive search strategy in the major medical databases, and selection and quality assessment performed by two independent reviewers
- It offers results on the impact of panel point-of-care tests, rather than just their accuracy
- Included studies were relatively small, and most notably for mortality, may be underpowered to detect clinically relevant differences between laboratory and point-of care tests.
- Statistical and clinical heterogeneity is evident within our meta-analysis, however the meta-analysis was considered carefully and heterogeneity reduced where possible by ensuring that studies of cardiac and general panel tests were not combined; moreover, we did not combine the before-after study (which also had a high risk of bias) with randomised controlled trials.
- Four studies excluded critically ill patients and patients with myocardial infarction, which may have biased mortality data.

For peer review only

For peer review only - http://bmjopen.bmj.com/site/about/guidelines.xhtml

Introduction

Background

All ambulatory care physicians frequently encounter diagnostic uncertainties in day-to-day practice. This can lead to missed opportunities for diagnoses or inappropriate referrals to secondary care. Patients with vague or non-specific symptoms can be the most challenging populations to assess.[1] Currently, most ambulatory care units use whole blood tests that are normally transported to and processed by a centralised clinical laboratory.

The technology behind in-vitro point-of-care testing (POCT) has developed extensively and the accuracy compared to standard methods for some tests is now established.[2,3] POCT now offers an alternative to conventional laboratory methods; it is performed on site, normally at the bedside and has a short turnaround time of typically 5-15 min.[4] POCT is being employed in a wide variety of healthcare settings and its use is predicted to expand dramatically.[5] Indeed, NHS England have stated that point-of-care tests will be available in urgent treatment centres in the UK from 2019.[6]

Importance

The use of POCT in ambulatory care has the potential to reduce diagnostic uncertainty and delay and physicians report that they would like to use these tests more, particularly to aid in the diagnosis of acute conditions.[7-9] It is expected that POCT facilitates healthcare processes such as the speed of discharge, leading to better use of healthcare resources, or enables quicker diagnosis and referral of patients with serious illness, which may lead to better patient outcomes. Panel tests are especially appealing in this patient group as they test multiple parameters simultaneously from the same finger prick of blood using the same platform, covering a range of conditions frequently found to cause acute presentations to ambulatory care. However, there are potential disadvantages associated with their implementation[10] and little is understood about the impact of POCT panels on day-to-day practice. Thus far, what is lacking is an up to date summary of all the available evidence on the impact of blood-based POCT panel testing in ambulatory care.

Objective

In this study, we performed a systematic review and meta-analysis to evaluate the quantitative impact of POCT in ambulatory care with a focus on blood-based panel tests.

Methods

This systematic review protocol has been registered in the PROSPERO International Prospective Register of Systematic Reviews (registration number: CRD42016035426). This protocol has been developed according to recommendations from the Cochrane Collaboration[11] and guidelines from the Preferred Reporting Items for Systematic Reviews and Meta-analyses statement[12] have been followed.

This systematic review forms part of a series of analyses from a larger overall review (in progress) which will assess the overall quantitative impact of all POCTs in ambulatory care, further subgroup analyses on CRP (C-reactive protein)[13] and influenza[14] have already been published.

Patient and Public Involvement

We consulted with an existing PPI panel of the NIHR Diagnostic Evidence Co-operative (DEC) Oxford specialising in research on in-vitro diagnostic technology, who have been involved in a number of previous

For peer review only - http://bmjopen.bmj.com/site/about/guidelines.xhtml

projects which incorporated POCT. They felt that this systematic review would be very important in defining the evidence for and against use of POCT in ambulatory care. One member described her experience as a patient in another European country where POCT for certain conditions was seen as part of standard care, and her surprise that this was not the case in the UK. They were specifically interested in the potential implications of POCT for facilitating earlier discharge from hospital.

Search strategy

We searched Ovid Medline (1946 to 2017), Embase (1974 to 2017), Cochrane Database of Systematic Reviews, Cochrane CENTRAL, Database of Abstracts of Reviews and Effects (DARE), Science Citation Index (1945-present) from inception. This systematic review forms part of a series of analyses from a larger overall review (in progress) which will assess the overall quantitative impact of all POCTs in ambulatory care. This main search was originally performed on 19th November 2015 and then updated on 21st March 2017, following this subgroup analyses on C-reactive protein (CRP)[13] and influenza[14] were published. Studies based in resource poor settings form another subgroup analyses from the overall review and will also be published separately. A further update was performed on October 22nd 2019 and was screened to identify papers that assessed the impact of blood-based panel tests in ambulatory care. We did not identify any new studies from this update, the PRISMA diagram in figure 1 summarises the process. A snowballing strategy was used to ensure that the search was as comprehensive as possible. We did not add a study design filter nor apply a language restriction. We performed citation searches of all full-text papers included in final review. The full strategy is included in appendix 1.

Selection of Studies

We included randomised controlled trials (RCTs), non-randomised but experimental and controlled studies including before-after studies. Included studies provided quantitative comparisons of the impact of blood-based POCT panel tests with laboratory testing and were based in ambulatory care.

Screening was divided between seven authors (CG, PST, JV, TA, JL, PT, AVB); two of these authors independently assessed the potential relevance of all titles and abstracts identified from the electronic search. Full-text papers of all potentially relevant papers were obtained and these were then further assessed by two of the authors. Conflicts were resolved by seeking the opinion of a third author and disagreements were discussed with the team to obtain consensus. The reason for excluding studies was recorded. The most common reasons for exclusion were studies that only focussed on diagnostic accuracy and did not consider impact. Another common reason was studies that only included qualitative comparisons or, if they did have quantitative data did not provide results on both the intervention and the control groups. We also excluded studies that evaluated POCT exclusively for monitoring purposes. We excluded panel tests that were not based on blood samples. Studies that included panel tests as part of a multifaceted intervention or combined blood-based panel tests with single tests or urine tests were also excluded. Systematic reviews were excluded with reference lists checked for potentially relevant studies for inclusion. For this subgroup analysis, appropriate studies were selected independently by two researchers.

Data extraction and quality assessment

Data extraction was performed by one author and independently checked by a second author. The authors extracted the following data from included studies: general study information (authors, title, publication year, study design and location/setting), inclusion and exclusion criteria and further information regarding the study population (to include mean age and severity of illness of participants). Details of the POCT intervention including which parameters were measured by the POCT device, details of the comparator

For peer review only - http://bmjopen.bmj.com/site/about/guidelines.xhtml

which was normally conventional blood test sent to laboratory; and finally outcomes assessed as listed below were also recorded.

The methodological quality of the included trials was assessed by two authors (AVB and CG and independently checked by TA). Any areas of conflict were discussed and resolved with a third member of the team where necessary. For RCTs we used the Cochrane Risk of Bias tool[11] including analysis of randomisation, allocation concealment, comparison of baseline characteristics and blinding. For non-randomised but experimental and controlled studies we used the Cochrane Risk of Bias tool plus an assessment of confounders[15] that were pre-specified and included assessing whether baseline characteristics were reported, whether they were similar in intervention and control groups and whether there was a detailed description of the usual care pathway.

Outcomes assessment

The primary outcome of interest was the impact of POCT on the time to decision regarding disposition i.e admission/referral termed disposition decision (DD) time. Secondary outcomes included length of stay (LOS) at the ambulatory care unit/practice and mortality. Hospital admission rates, rates of repeat attendance after discharge / re-admission were also examined.

Statistical analyses

Individual study estimates were pooled in a meta-analysis using random-effects inverse-variance model, and study-to-study heterogeneity was assessed using the I^2 test statistic in combination with visual inspection using Review Manager.[16] We used mean differences in DD and LOS time (minutes) and their corresponding 95% confidence intervals (95% CI). Where studies reported the median time to disposition decision and length of stay, attempts were made to contact the original authors for mean times and standard deviations. In the case where they were not available, we estimated them using an approach suggested by Wan et al.[17] which approximates reported medians and quartiles/ranges to corresponding mean and standard deviations robustly by also taking into account studies' sample sizes to avoid small study bias.

Results

Description of included studies

The combined total of the original search and update was 28,160 studies as summarized in PRISMA[18] diagram below (**figure 1**). Nine studies relevant to POCT blood-based panel tests were selected and reported here, including eight RCTs and one before-after study. Seven studies reported on general panel tests and two studies focused on cardiac panels.

In total, 19,562 participants were included (see study characteristics, **table 1**). The majority of participants were either adults or defined as being aged over 15 years, with the exception of one study,[19] which recruited from a paediatric emergency departments (ED) where all participants were aged under 21 years. All the studies were based in ED departments except one study[20] that was based in the Canadian ambulance service. Notably, there were no studies based in primary care that focused on panel testing for diagnosis in the acute setting, there were only studies that monitored patients with chronic disease or analysed single tests.

A variety of different POCT panel devices were used in the studies. Although there was variability regarding the specific tests performed by different devices, general panels always included basic metabolic

For peer review only - http://bmjopen.bmj.com/site/about/guidelines.xhtml

Study	Setting	Device (Manufacturer)	Tests performed	Participant characteristics	Sample size
**Parvin* 1996**[27]	ED USA	i-STAT (i-STAT Corp, Princeton, NJ)	Sodium, potassium, chloride, blood urea nitrogen, glucose, haematocrit, haemoglobin	Patients presenting to the ED between Dec 1994-Jan 1995 (control period 1), Feb-April 1995 (intervention period) and April 1995 (control period 2) and who had blood tests done that were available on the i-STAT. Only 5.3% of patients had no other central laboratory testing performed in addition to i-STAT	2067
**Kendall 1998**[28]	ED<br>UK	i-STAT (Abbott)<br>2 cartridges evaluated	Sodium, potassium, chloride, urea, glucose, packed cell volume (PCV), calculates Hb from PCV<br>pH, partial pressure carbon dioxide (ppCO2), partial pressure oxygen (ppO2), bicarbonate, total CO2, base excess, oxygen saturation	Representative sample of adult ED patients who needed blood tests.<br>No exclusion criteria.	1728

parameters such as sodium, potassium and glucose. Creatinine and basic blood gas analysis such as total carbon dioxide and base excess were also commonly featured. Cardiac panels always included troponin in combination with BNP[20] or creatine kinase (myocardial type) and myoglobin.[21]

There was variation in participant inclusion criteria. Two studies included a representative sample of adult ED patients who needed blood tests,[22,23] and one included patients "whose physicians ordered a comprehensive metabolic panel."[24] Two studies randomized all patients seen in ED but limited inclusion to the trial to only those patients whose blood work fell entirely within capabilities of the POCT devices used.[19,25] Only one study[26] recorded data on the number of patients who also required tests that were beyond the scope of the POCT device. Personal communication to authors was attempted to obtain this data from the other studies but it had either not been recorded or authors did not respond. Two studies excluded patients who required critical care.[19,24] The cardiac panel studies were more specific in their inclusion criteria, including only patients with chest pain and/or dyspnoea[20] and also had more extensive exclusion criteria such as patients with myocardial infarction on ECG.[21]

Figure 2, summarises the key features of methodological assessment for the 8 included RCTs and the risk of bias for the before-after study is available in **appendix 2**. In general, for the included RCTs methodological quality was variable, with the exception of one study[26] being at high risk or unclear for most domains. The before-after study[27] also assessed as 'high or 'unclear' on all domains; it was also 'high risk' for the confounders assessment as neither the baseline characteristics of participants nor the care pathway for the control group were described in detail.

For peer review only - http://bmjopen.bmj.com/site/about/guidelines.xhtml

**Murray 1999**[25]	ED<br>Canada	NOVA 16 CRT™ Spectral™ Cardiac STATus Test Kit (Nova Biomedical)	Creatinine, sodium, potassium, chloride, total CO2, glucose, blood urea nitrogen, haematocrit, qualitative creatine kinase MB isoenzyme (CK-MB), and myoglobin	Adult ED patients, all patients seen in ED randomized but only those patients **whose blood work fell entirely within capabilities of POCT** were selected.	180
**Hsiao 2007**[19]	Tertiary paediatric ED<br>USA	i-STAT (Abbott)	Sodium, potassium, chloride, bicarbonate, glucose, blood urea nitrogen, creatinine, ionized calcium, haematocrit, basic blood gas analysis	Paediatric ED patients aged under 21 years old **whose blood work fell entirely within capabilities of POCT.** Critically ill patients excluded.	239
**Lee 2011**[23]	Multicentre: 5 EDs<br>South Korea	Piccolo xpress® (Abbott) Piccolo Metabolic Reagent Discs	Protein, albumin, alk phos, alanine aminotransferase, aspartate aminotransferase, nitrogen, calcium, cr, glucose, postasium, sodium, bilirubin, total CO2	ED patients aged 15 years and older clinically required to have chemistry laboratory tests.	2323
**Illahi 2012**[26]	ED<br>UK	Xpand Plus analyser (Siemens) XS 1000 analyser (Sysmex)	Albumin, Alkaline phosphatase, Amylase, Bilirubin, Calcium, Creatinine, CRP, glucose, paracetamol, phosphate, potassium, sodium, urea, FBC, WBC and differential	Adult ED patients. Some samples then underwent further testing in the central laboratory if required tests were not available from POCT device.	47
**Jang 2013**[24]	ED<br>Korea	Piccolo xpress® (Abbott)<br>Piccolo Metabolic Reagent Discs	Total protein, albumin, alkaline phosphatase, alanine aminotransferase, aspartate aminotransferase, urea, nitrogen, calcium, chloride, creatinine, glucose, potassium, sodium, total bilirubin and total carbon dioxide	ED patients aged 15 years and older whose physicians ordered a comprehensive metabolic panel.<br>Critically ill patients excluded.	10244
**Goodacre** 2011**[21]	Multicentre 6 EDs UK	Stratus CS (Siemens) Cardiac analyser panel	CK, myocardial type, myoglobin, troponin 1	Adult ED patients with chest pain. Several exclusion criteria applied including patients with ECG changes consistent with myocardial infarction/high risk acute coronary syndrome, confirmed or suspected serious non-coronary pathology.	2243
**Ezekowitz** 2014**[20]	Ambulance Service<br>Canada	Cardio2 panel (Alere)	Troponin and B-type natriuretic peptide (BNP)	Adults > 18 years of age who activated emergency medical services (EMS) for acute chest discomfort or dyspnea for which	491

For peer review only - http://bmjopen.bmj.com/site/about/guidelines.xhtml

(Out of hospital)		acute cardiovascular disease was deemed to be the most probable diagnosis. Patients excluded if ST-elevation on ECG and non-cardiovascular cause suspected / recurrent dyspnea.

Table 1: Characteristics of included studies, setting, device, tests performed, patient characteristics, sample size

*All studies were RCTs except from Parvin 1996(26) which was a before-after study design
** Assessed cardiac panels

Primary Outcome

Disposition Decision Time

The DD time, was specifically reported in 3 studies.[14,19,26] As summarized in **figure 3**, POCT reduced the overall DD time by 39 minutes (95% CI -42.2 to -36.6, I^2=0%)) compared to usual care. This reduction was increased to 48 minutes in patients who did not require additional laboratory tests (95% CI -61.11 to -34.05, I^2 =0%). Hsiao et al[19] recruited only from paediatric ED (patients aged under 21 years) whilst the other studies included adult patients. These all evaluated blood-based panel POCT devices in general ED patients, but Hsiao only reports results for patients whose blood work fell entirely within the capabilities of the POCT device, where as Illahi et al[26] report these different subgroups of patients separately. Illahi et al[26] reported point estimates as average values and this has been taken as median values in our analysis, attempts were made to contact the author for confirmation but this was not successful. Sensitivity analysis excluding Illahi[26], demonstrated robust findings (**appendix 3**). Kendall et al[22] did not specifically measure DD so their results were not included in the meta-analysis. However they did describe that decisions regarding the management plan were made 74 minutes earlier (95% CI 68 min to 80 min, p<0.0001) when POCT was used for haematological tests as compared to central laboratory testing and 86 minutes earlier (80 min to 92 min p<0.0001) for biochemical tests.

Secondary outcomes

Length of Stay

LOS in ED was measured in six studies.[19,21,22,24,25,27] Four of these studies[19,22,24,25] were RCTs that assessed general POCT panel tests in ED and these were combined in the meta-analysis, summarised in **figure 4**. These included three studies with adult participants[22,24,25] and one study[19] based in paediatric ED. A significant reduction in ED LOS of 33 minutes (95% CI -60.66 to -5.84) was observed in the POCT group although wide 95% confidence intervals were noted (**figure 4**). This reduction was increased to 37 minutes (95% CI -53.08 to -21.77) in patients who only required POCT (and needed no additional laboratory tests). Three of these studies[19,24,25] provided further specific data on the LOS for patients who were admitted and discharged. This data was combined in **figure 5**, POCT was found to reduce the overall LOS for patients who were later discharged by 34 minutes (95% CI -63.68 to -5.16) although wide CI were noted. There was no statistically significant difference between LOS in POCT versus usual care in patients who were later admitted (**figure 5**). In their before-after study of 4985 patients Parvin et al[27] evaluated a general POCT panel in ED; median LOS with POCT was 209 minutes (111 to 368) versus 201 (106-345) for usual care which was not statistically significant. Subgroup analysis by presenting symptoms and discharge/admit status did not detect any further differences.

For peer review only - http://bmjopen.bmj.com/site/about/guidelines.xhtml

LOS in ED was also measured in two studies on POCT cardiac panels.[20,21] One study integrated POCT into emergency medical services in Canada[20] and assessed patients with chest pain or dyspnoea, they found no difference in time from first medical contact to final disposition (9.2 (95% CI 7.3-11.1) hours for the POCT group and 8.8 (95% CI 6.3-12.1) hours for usual care (P=0.609). Goodacre et al[21] recorded successful discharge home from ED for patients with chest pain which they defined as having left hospital (or awaiting transport) within 4 hours of arrival and no adverse events occurring over the next 3 months. POCT cardiac biomarker panels were associated with an increased rate of successful discharge (32% vs 13% in the usual care group, OR 3.81, 95% CI 3.01-4.82; p<0.001), although analysis of the original data demonstrated that the median LOS in ED for the POCT group was longer at 216 minutes (IQR 179-238) compared to the usual care pathway of 188 minutes (IQR 142-225).

Mortality

Three studies included data on patient mortality.[20,21,22] There was no significant difference in mortality between POCT and laboratory testing as demonstrated in **figure 6**. Two of these studies evaluated cardiac panels, calculated risk ratios of death were 2.98 (0.60 to 14.74)[21] and 0.80 (0.22 to 2.94),[20] one study on general panels reported a relative risk of death of 1.16 (0.79 to 1.68).[22]

For the other secondary outcomes, only one study reported hospital admission rates[28] and found that this was not significantly different between the POCT and laboratory groups (difference 1.7, CI -1.7,5.1 P=0.33). Rates of repeat ED attendance after discharge and re-hospitalization were also recorded by Ezekowitz et al[20] and there was no significant difference detected between POCT and laboratory testing (P=0.320, P=0.712, respectively).

In terms of exploratory outcomes, there is evidence that unwell patients benefited from faster decision making with POCT. Kendall et al[22] describe how 59 out of 859 POCT patients had changes in their management in which timing was considered to be critical; these included decision to intubate/ventilate. POCT was also associated with reduced time to CT from ED arrival,[24] with a median difference of 11 minutes (95% CI 3 to 19).

Discussion

Statement of principal findings

This systematic review found that general panel tests performed at the point-of-care may result in faster disposition and management decisions, which in turn might reduce LOS for patients who are subsequently discharged from the ED. This is not associated with changes in mortality. There is also no gain in LOS for patients who are admitted to hospital. These results perhaps suggest that specific groups of patients may benefit from the introduction of POCT in an ED setting; such as, well patients who could be discharged faster and unwell patients who need critical interventions more quickly. The LOS advantage was attenuated when extra tests were required from the laboratory in addition to the POCT panel.

Strengths and weaknesses of the study

This study was conducted robustly, using a comprehensive search strategy in the major medical databases, and selection and quality assessment performed by two independent reviewers. It offers results on the impact of panel POCT, rather than just their accuracy. A comprehensive search strategy also brings limitations, variation in countries, healthcare practices and usual care may have contributed to high

For peer review only - http://bmjopen.bmj.com/site/about/guidelines.xhtml

For peer review only

heterogeneity for some outcomes. Another consideration is that we present here studies published from 1996 to 2014 and clinical practice will have changed during this time, moreover it is concerning that no new impact evaluations of blood-based panels have been performed in the last five years despite the increase in implementation of POCT. Although impact studies are an integral part of the evidence cycle for new tests,[28] they are also difficult to organize and subject to bias. In our review, blinding clinicians and patients from the intervention was not possible by nature, introducing a risk of bias and in general most studies were not blinded by outcome assessment. Only a small number of relatively small studies were included in this meta-analysis which did not allow further exploration of small study effect. Moreover, most notably for mortality, the results may suffer from being underpowered to detect differences between POCT and laboratory testing.

Statistical and clinical heterogeneity is evident within our meta-analysis, particularly for LOS results. The meta-analysis was considered carefully and reduced where possible by ensuring that studies of cardiac and general panels tests were not combined; moreover, we did not combine the before-after study (which also had a high risk of bias)[27] with RCTs. Multiple factors influence our primary and secondary outcomes and these variables are responsible for much of the clinical heterogeneity. It is important to consider the system in which POCT is implemented and which ED triage systems are used. For example, if blood tests are requested on arrival in ED than laboratory results might be available at the time of physician review anyway and thus there would be fewer benefits to POCT. Moreover, practicalities such as how quickly radiology is available and how samples are transported to laboratories will impact results significantly.

There are also many factors which impact ED LOS specifically, especially availability of inpatient beds and this may be the reason for the reduced benefit on LOS in the admitted group. Other important factors that differed between the studies and between different hospitals,[21] included the time of day that POCT was available; with one study only performing POCT during working hours, as well as the availability and seniority of clinical staff. Furthermore, the studies differed in their inclusion criteria, and as demonstrated by our subgroup analysis, the benefits of POCT on LOS was proportional to the spectrum of tests available. This perhaps explains why Parvin et al[27] did not demonstrate any benefit in reduced LOS from POCT as 95% of these patients also required additional laboratory tests in addition to the POCT panel.[28] Moreover, there is further evidence of this association from other studies that combined single and multiple tests and demonstrated a significant reduction in LOS for POCT.[29,30]

Other factors relate specifically to study protocol, for example Goodacre et al[21] describe how the LOS was longer for the POCT because POCT patients did not leave the ED until their POCT testing (at baseline and 90 minutes) was complete, whereas the standard care group could leave the ED as soon as medical assessment was complete and a decision to admit (or discharge in a few cases) had been made. Therefore, the POCT group spent longer in the ED but were more likely to go home before the 4 hour point, whilst the usual care group spent less time in the ED because they were more likely to be admitted to a ward (and thus leave the ED) at any earlier time (personal communication with author).

An important limitation to highlight is that four studies excluded critically ill patients [19,24] and patients with myocardial infarction,[20,21] which may have biased mortality data.

Comparison with other studies

The benefit of POCT in ambulatory patients has been shown by Kankaanpaa et al,[31] where single and panel tests were implemented in ambulatory patients presenting to a Finnish ED who also see primary care patients outside of office hours. They excluded all patients who were admitted to hospital. Median LOS in the control phase was 3.51 hours (3.38-4.04) and this was reduced to 3.22 hours (3.12-3.31 p=0.000) with the implementation of POCT; moreover, the combination of POCT with an early assessment triage model, reduced LOS to 3.05 hours (02.59-03.12, p=0.033). This study[31] appropriately recorded which patients also

For peer review only - http://bmjopen.bmj.com/site/about/guidelines.xhtml

required additional laboratory testing and found that this was lowest when POCT and an early assessment triage model were combined, when 68% of patients did not require additional blood tests (which was also associated with the greatest reduction in LOS).

Lingervelder[32] et al performed a systematic review to assess POCT implementation aspects addressed in primary care. They found that only 8% of evaluations included measurement of clinical utility, even though GPs perceive this as the most important issue to consider. They found that the most frequently evaluated tests were single tests such as HbA1c, CRP and D-dimer so these would not have been included in our panels review.

Implications for research and practice

Future research is required to understand the impact that POCT panels have in assisting with the decision to admit or discharge patients and analyse their cost-effectiveness.[33,34] We would recommend that future trials assess successful discharge, rate of admission and rate of adverse events rather than just focussing on time to discharge or disposition decision. Moreover, the relationship between ED overcrowding and LOS needs to be better understood as reductions in LOS do not necessarily reduce overcrowding.[35] This review suggests that there are specific subgroups that may benefit most from the implementation of POCT, and future studies should focus on these groups and establish which tests should be combined in a POCT panel such as CRP.

Theoretically there are also advantages to using POCT in the primary care setting. For example, it may help to identify acute kidney injury or atypical presentation of myocardial infarction. However, it may not be time efficient or cost effective. As none of the included studies were based in primary care, understanding the impact of POCT in this setting remains a research priority. Research outcomes and study designs in this environment need to be carefully considered, particularly as laboratory testing may not be available at all or maybe delayed, particularly regarding home visits and for patients in rural areas.

It is important to understand how POCT changes management decisions particularly regarding admission and to monitor whether the thresholds for ordering tests changes with POCT implementation.[34] Future research should also consider patients views on POCT and how implementation could be linked to digital transformations to maximise benefits for patients and clinicians alike.

There is a clear gap between evidence and policy which needs to be addressed. NHS England have stated that the POCT i-STAT will be available in urgent treatment centres in the UK from 2019[6] but there is currently a lack of evidence to underpin this. Future research needs to based in ambulatory settings and should identify how POCT can contribute meaningful changes to patient care rather than simply examining health care processes and must focus on the impact of POCT implementation.

Copyright: I, Clare R Goyder, the Submitting Author has the right to grant and does grant on behalf of all authors of the Work (as defined in the below author licence), an exclusive licence and/or a non-exclusive licence for contributions from authors who are: i) UK Crown employees; ii) where BMJ has agreed a CC BY licence shall apply, and/or iii) in accordance with the terms applicable for US Federal Government officers or employees acting as part of their official duties; on a worldwide, perpetual, irrevocable, royalty-free basis to BMJ Publishing Group Ltd ("BMJ") its licensees and where the relevant Journal is co-owned by BMJ to the co-owners of the Journal, to publish the Work in EMJ and any other BMJ products and to exploit all rights, as set out in our licence.

Data availability: All data relevant to the study are included in the article or uploaded as supplementary information

For peer review only - http://bmjopen.bmj.com/site/about/guidelines.xhtml

Contributors: TA designed the initial search strategy. Screening was carried out by JV, JL, CG, PST, TA, PT, AV. Data extraction was performed by CG, AV and TA. CG and PST carried out the meta-analysis with advice from AV and GH. CG and AV drafted the manuscript. All authors commented and co-drafted the final version of this article. CG is the guarantor and affirms that the manuscript is an honest, accurate, and transparent account of the study being reported; that no important aspects of the study have been omitted; and that any discrepancies from the study as planned (and, if relevant, registered) have been explained.

Funding: This paper presents independent research funded by the National Institute for Health Research School for Primary Care Research (NIHR SPCR), funding round 11, award number 309. CG is a Wellcome Trust Doctoral Fellow [grant number 203921]. JV, PT, GH and AV are supported through the National Institute for Health Research (NIHR) Community Healthcare MedTech and IVD Co-operative Oxford at Oxford Health Foundation Trust (award number MIC-2016-018). JL is an NIHR In-Practice Fellow.

Disclaimer: The views expressed are those of the author(s) and not necessarily those of the NIHR, the NHS or the Department of Health

Competing interests: All authors have completed the Unified Competing Interest form (available on request from the corresponding author) and declare: no support from any organisation for the submitted work [or describe if any]; no financial relationships with any organisations that might have an interest in the submitted work in the previous three years [or describe if any], no other relationships or activities that could appear to have influenced the submitted work [or describe if any].

Ethical approval: Not required.

Systematic review registration: PROSPERO CRD42016035426

Acknowledgements: We thank Nia Roberts, Dr Jennifer Hirst and Dr José M. Ordóñez-Mena for their assistance with this project.

For peer review only

Figure legends:

Figure 1: PRISMA flow diagram

Figure 2: Risk of bias summary for included randomised controlled trials

Figure 3: Forest plot of comparison of time to disposition decision in minutes for patients who needed laboratory testing in addition to POCT and for patients whose blood work fell entirely within the capabilities of POCT

Figure 4: Forest plot of comparison of length of stay time in minutes for patients who needed laboratory testing in addition to POCT and for patients whose blood work fell entirely within the capabilities of POCT

Figure 5: Forest plot of comparison of length of stay time in minutes for patients who underwent POCT testing versus laboratory testing, split into subgroups for patients who were admitted/discharged

Figure 6: Relative risk of death in POCT compared to laboratory testing for general and cardiac panel tests performed in ED, and cardiac panels tested by the ambulance service

For peer review only - http://bmjopen.bmj.com/site/about/guidelines.xhtml

References

1. Kostopoulou O, Delaney BC, Munro CW. Diagnostic difficulty and error in primary care - A systematic review. Vol. 25, Family Practice. 2008. p. 400–13.
2. Verbakel JY, Aertgeerts B, Lemiengre M, Sutter A De, Bullens DMA, Buntinx F. Analytical accuracy and user-friendliness of the Afinion point-of-care CRP test. J Clin Pathol [Internet]. 2014;67(1):83–6. Available from: http://jcp.bmj.com/lookup/doi/10.1136/jclinpath-2013-201654
3. Luukkonen AAM, Lehto TM, Hedberg PSM, Vaskivuo TE. Evaluation of a hand-held blood gas analyzer for rapid determination of blood gases, electrolytes and metabolites in intensive care setting. Clin Chem Lab Med. 2016;54(4):585–94.
4. Abel G. Expert Review of Molecular Diagnostics Current status and future prospects of point-of-care testing around the globe. Expert Rev Mol Diagn [Internet]. 2015;15(7):853–5. Available from: http://www.tandfonline.com/action/journalInformation?journalCode=iero20
5. Sohn AJ, Hickner JM, Alem F. Use of Point-of-Care Tests (POCTs) by US Primary Care Physicians. J Am Board Fam Med [Internet]. 2016;29(3):371–6. Available from: http://www.jabfm.org/cgi/doi/10.3122/jabfm.2016.03.150249
6. NHS England Urgent Treatment Centres [Internet]. 2017. Available from: https://www.england.nhs.uk/wp-content/uploads/2017/07/urgent-treatment-centres–principles-standards.pdf [Accessed 7th June 2018]
7. Cals JWL, Schols AMR, van Weert HCPM, Stevens F, Zeijen CGIP, Holtman G, et al. [Point-of-care testing in family practices: present use and need for tests in the future]. Ned Tijdschr Geneeskd [Internet]. 2014;158:A8210. Available from: http://www.ncbi.nlm.nih.gov/pubmed/25515388
8. Turner PJ, Van den Bruel A, Jones CHD, Plüddemann A, Heneghan C, Thompson MJ, et al. Point-of-care testing in UK primary care: A survey to establish clinical needs. Fam Pract. 2016;33(4):388–94.

For peer review only - http://bmjopen.bmj.com/site/about/guidelines.xhtml

9. Howick J, Cals JWL, Jones C, Price CP, Pluddemann A, Heneghan C, et al. Current and future use of point-of-care tests in primary care: an international survey in Australia, Belgium, The Netherlands, the UK and the USA. BMJ Open [Internet]. 2014;4(8):e005611–e005611. Available from: http://bmjopen.bmj.com/cgi/doi/10.1136/bmjopen-2014-005611
10. Gutierres SL, Welty TE. Point-of-Care Testing: An Introduction. Vol. 38, Annals of Pharmacotherapy. 2004. p. 119–25.
11. Higgins JP, Green S. Cochrane Handbook for Systematic Reviews of [Internet]. Vol. Version 5., The Cochrane Collaboration. 2008. Version 5.1.0. Available from: http://onlinelibrary.wiley.com/doi/10.1002/9780470712184.fmatter/summary
12. Moher D, Liberati A, Tetzlaff J, Altman DG, The PRISMA Group (2009) Preferred Reporting Items for Systematic Reviews and Meta-Analyses: The PRISMA Statement. PLoS Med 6(7): e1000097. https://doi.org/10.1371/journal.pmed.1000097
13. Verbakel JY, Lee JJ, Goyder CR, Tan PS, Ananthakumar T, Turner PJ, l Hayward G, Van den Bruel A. The impact of point-of-care c-reactive protein in ambulatory care: A systematic review and meta-analysis. BMJ Open. 2019; 1:9 (1):e025036
14. Lee JJ, Verbakel JY, Goyder CR, Ananthakumar T, Tan PS, Turner PJ, Hayward G, Van den Bruel A. Clinical Infectious Diseases. 2018; ciy837, https://doi.org/10.1093/cid/ciy837
15. Reeves C B, Deeks J J, Higgins P.,T. J, Wells A G. Cochrane Handbook: Special Topics: Ch 13: Including non-randomized studies. In: Cochrane Handbook for: Systematic Reviews of Interventions [Internet]. 2011. p. 389–432. Available from: http://training.cochrane.org/handbook [Accessed 7th June 2018)
16. Review Manager (RevMan). No Title. Review Manager (RevMan) Version 5.3. Copenhagen: The Nordic Cochrane Centre, The Cochrane Collaboration, 2014.
17. Wan X, Wang W, Liu J, Tong T. Estimating the sample mean and standard deviation from the sample size, median, range and/or interquartile range. BMC Med Res Methodol. 2014;14(1).
18. Prisma Flow Diagram [Internet]. Available from: http://prisma-statement.org/prismastatement/flowdiagram.aspx [accessed 13th June 2018]
19. Hsiao AL, Santucci KA, Dziura J, Baker MD. A randomized trial to assess the efficacy of point-of-care testing in decreasing length of stay in a pediatric emergency department. Pediatr Emerg Care [Internet]. 2007;23(7):457–62. Available from: http://www.ncbi.nlm.nih.gov/pubmed/17666926
20. Ezekowitz JA, Welsh RC, Gubbels C, Brass N, Chan M, Keeble W, et al. Providing rapid out of hospital acute cardiovascular treatment 3 (PROACT-3). Can J Cardiol. 2014;30(10):1208–15.
21. Goodacre SW, Bradburn M, Cross E, Collinson P, Gray A, Hall AS. The Randomised Assessment of Treatment using Panel Assay of Cardiac Markers (RATPAC) trial: A randomised controlled trial of point-of-care cardiac markers in the emergency department. Heart. 2011;97(3):190–6.
22. Kendall J, Reeves B, Clancy M. Point of care testing: randomised controlled trial of clinical outcome. BMJ. 1998;316(7137):1052–7.
23. Lee EJ, Shin S Do, Song KJ, Kim SC, Cho JS, Lee SC, et al. A point-of-care chemistry test for reduction of turnaround and clinical decision time. In: American Journal of Emergency Medicine. 2011. p. 489–95.
24. Jang JY, Shin S Do, Lee EJ, Park CB, Song KJ, Singer AJ. Use of a comprehensive metabolic panel point-of-care test to reduce length of stay in the emergency department: A randomized controlled trial. Ann Emerg Med. 2013;61(2):145–51.
25. Murray RP, Leroux M, Sabga E, Palatnick W, Ludwig L. Effect of point of care testing on length of stay in an adult Emergency Department. J Emerg Med. 1999;17(5):811–4.
26. Illahi MN, Lapworth R, Bates P. The effect of point of care testing on clinical decision making in an emergency department [Internet]. Vol. 11, Journal of the Liaquat University of Medical and Health Sciences. 2012: 153–7.
27. Parvin CA, Lo SF, Deuser SM, Weaver LG, Lewis LM, Scott MG. Impact of point-of-care testing on patients' length of stay in a large emergency department. Clin Chem. 1996;42(5):711–7.
28. Verbakel JY, Turner PJ, Thompson MJ, Plüddemann A, Price CP, Shinkins B, et al. Common evidence

For peer review only - http://bmjopen.bmj.com/site/about/guidelines.xhtml

gaps in point-of-care diagnostic test evaluation: A review of horizon scan reports. BMJ Open. 2017;7(9).

29. Lee-Lewandrowski E, Corboy D, Lewandrowski K, Sinclair J, McDermot S, Benzer TI. Implementation of a point-of-care satellite laboratory in the emergency department of an academic medical center: Impact on test turnaround time and patient emergency department length of stay. Arch Pathol Lab Med. 2003;127(4):456–60.
30. Asha SE, Chan ACF, Walter E, Kelly PJ, Morton RL, Ajami A, et al. Impact from point-of-care devices on emergency department patient processing times compared with central laboratory testing of blood samples: a randomised controlled trial and cost-effectiveness analysis. Emerg Med J [Internet]. 2014 Sep 1 [cited 2018 Mar 7];31(9):714–9. Available from: http://www.ncbi.nlm.nih.gov/pubmed/23748157
31. Kankaanpaa M, Raitakari M, Muukkonen L, Gustafsson S, Heitto M, Palomaki A, et al. Use of point-of-care testing and early assessment model reduces length of stay for ambulatory patients in an emergency department. Scand J Trauma Resusc Emerg Med [Internet]. 2016;24(1):125. Available from: http://dx.doi.org/10.1186/s13049-016-0319-z
32. Lingervelder D, Koffijberg H, Kusters R, IJzerman MJ. Point-of-care testing in primary care: A systematic review on implementation aspects addressed in test evaluations.Int J Clin Pract. 2019 Oct;73(10):e13392. doi: 10.1111/ijcp.13392. Epub 2019 Aug 1933.
33. Fermann GJ, Suyama J. Clinical Laboratory in Emergency Medicine Point of care testing in the emergency department Available from: https://ac.els-cdn.com/S0736467902004298/1-s2.0-S0736467902004298-main.pdf?_tid=63c3e414-0592-4cc5-a81b-37abed7224f9&acdnat=1520427388_2833d860886622e640aa71267a9157dd
34. Tsai WW, Nash DB, Seamonds B, Weir GJ. Point-of-care versus central laboratory testing: an economic analysis in an academic medical center. Clin Ther [Internet]. 1994 [cited 2018 Mar 26];16(5):898–910; discussion 854. Available from: http://www.ncbi.nlm.nih.gov/pubmed/7859247
35. Goodacre SW. The Practice Of Emergency Medicine/Editorial. Point-of-Care Testing May Reduce Length of Stay but Not Emergency Department Crowding. Ann Emerg Med [Internet]. 2012 [cited 2018 Mar 7];61:153–4. Available from: http://dx.doi.org/10.1016/j.annemergmed.2012.09.001

For peer review only

For peer review only - http://bmjopen.bmj.com/site/about/guidelines.xhtml

For peer review only

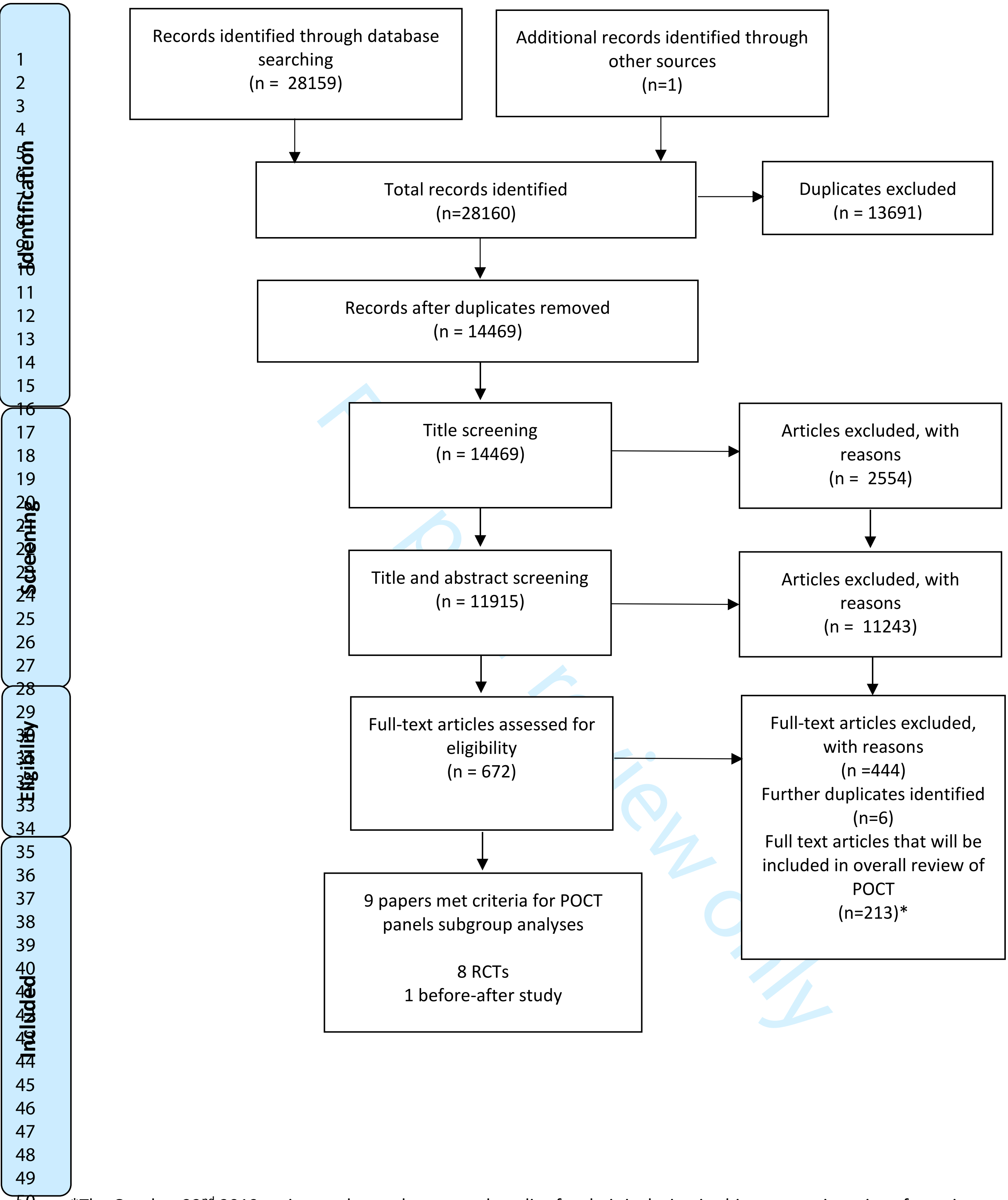

*The October 22nd 2019 review update only screened studies for their inclusion in this systematic review, focussing on the impact of point-of-care panel tests in ambulatory care, and did not assess suitability for inclusion in the overall review. The 213 articles currently included in the overall POCT review is correct up to the previous update on 17th March 2017

	Random sequence generation (selection bias)	Allocation concealment (selection bias)	Blinding of participants and personnel (performance bias)	Blinding of outcome assessment (detection bias)	Incomplete outcome data (attrition bias)	Selective reporting (reporting bias)	Other bias
Ezekowitz 2014	+	?	?	?	+	+	?
Goodacre 2011	+	+	−	−	+	+	−
Hsiao 2007	+	+	−	−	+	+	−
Illahi 2012	?	?	−	?	−	?	−
Jang 2013	+	+	?	−	+	+	+
Kendall 1998	+	+	?	?	+	+	−
Lee 2011	+	?	?	−	−	+	?
Murray 1999	+	−	?	−	?	+	?

For peer review only - http://bmjopen.bmj.com/site/about/guidelines.xhtml

Study or Subgroup	POC Mean	POC SD	POC Total	Lab Mean	Lab SD	Lab Total	Weight	Mean Difference IV, Random, 95% CI
**3.1.1 Patients who needed laboratory tests in addition to POCT**								
Lee 2011	48.5	22.7	616	88	27.5	609	95.3%	-39.50 [-42.33, -36.67]
Illahi 2012	137	59.3	18	166	67.61	29	0.6%	-29.00 [-65.82, 7.82]
**Subtotal (95% CI)**			**634**			**638**	**95.8%**	**-39.44 [-42.26, -36.62]**
Heterogeneity: Tau² = 0.00; Chi² = 0.31, df = 1 (P = 0.58); I² = 0%								
Test for overall effect: Z = 27.44 (P < 0.00001)								
**3.1.2 Patients whose blood work fell entirely within capabilities of POCT**								
Hsiao 2007	93.1	59.5	114	138.3	52.6	111	3.5%	-45.20 [-59.86, -30.54]
Illahi 2012	104.8	40.4	10	166	67.6	29	0.6%	-61.20 [-96.30, -26.10]
**Subtotal (95% CI)**			**124**			**140**	**4.2%**	**-47.58 [-61.11, -34.05]**
Heterogeneity: Tau² = 0.00; Chi² = 0.68, df = 1 (P = 0.41); I² = 0%								
Test for overall effect: Z = 6.89 (P < 0.00001)								
**Total (95% CI)**			**758**			**778**	**100.0%**	**-39.78 [-42.53, -37.02]**
Heterogeneity: Tau² = 0.00; Chi² = 2.32, df = 3 (P = 0.51); I² = 0%								
Test for overall effect: Z = 28.27 (P < 0.00001)								
Test for subgroup differences: Chi² = 1.33, df = 1 (P = 0.25), I² = 24.9%								

Mean Difference
IV, Random, 95% CI

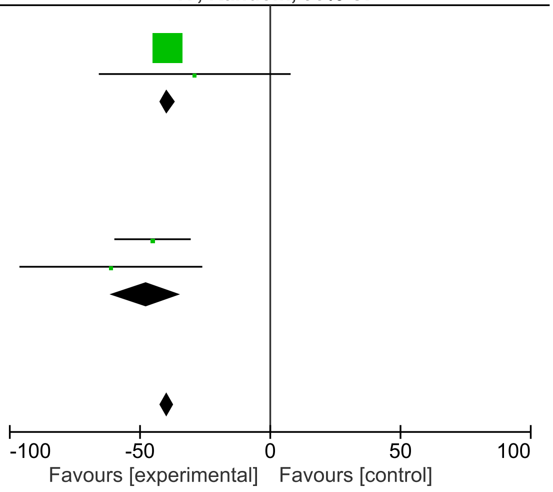

For peer review only

	Experimental			Control				Mean Difference
**Study or Subgroup**	**Mean**	**SD**	**Total**	**Mean**	**SD**	**Total**	**Weight**	**IV, Random, 95% CI**
**4.1.1 Patients who needed laboratory tests in addition to POCT**								
Jang 2013	519.3	590.2	5154	579.7	691.8	5090	24.3%	-60.40 [-85.32, -35.48]
Kendall 1998	188	97.2	859	193	105.2	868	29.3%	-5.00 [-14.55, 4.55]
**Subtotal (95% CI)**			**6013**			**5958**	**53.6%**	**-31.46 [-85.69, 22.78]**
Heterogeneity: Tau² = 1441.90; Chi² = 16.56, df = 1 (P < 0.0001); I² = 94%								
Test for overall effect: Z = 1.14 (P = 0.26)								
**4.1.2 Patients whose blood work fell entirely within capabilities of POCT**								
Hsiao 2007	190.2	63.8	114	228	67.6	111	27.2%	-37.80 [-54.98, -20.62]
Murray 1999	228.7	137.1	93	264.3	122.9	87	19.2%	-35.60 [-73.59, 2.39]
**Subtotal (95% CI)**			**207**			**198**	**46.4%**	**-37.43 [-53.08, -21.77]**
Heterogeneity: Tau² = 0.00; Chi² = 0.01, df = 1 (P = 0.92); I² = 0%								
Test for overall effect: Z = 4.68 (P < 0.00001)								
**Total (95% CI)**			**6220**			**6156**	**100.0%**	**-33.25 [-60.66, -5.84]**
Heterogeneity: Tau² = 643.29; Chi² = 24.16, df = 3 (P < 0.0001); I² = 88%								
Test for overall effect: Z = 2.38 (P = 0.02)								
Test for subgroup differences: Chi² = 0.04, df = 1 (P = 0.84), I² = 0%								

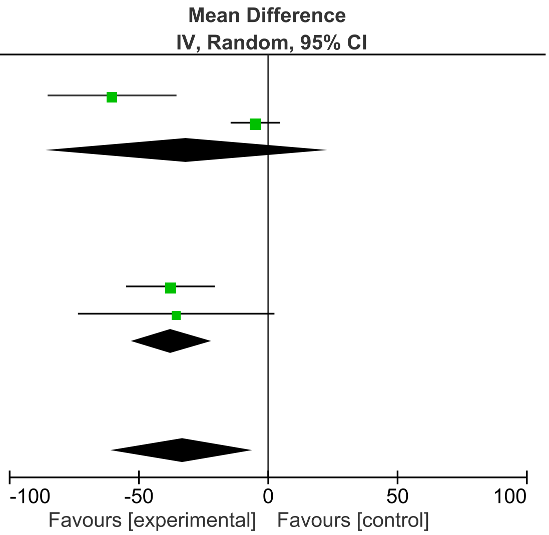

For peer review only

Study or Subgroup	Experimental Mean	Experimental SD	Experimental Total	Control Mean	Control SD	Control Total	Weight	Mean Difference IV, Random, 95% CI
**5.1.1 Admitted LOS**								
Hsiao 2007	207	85	35	237.5	82.5	48	50.0%	-30.50 [-67.07, 6.07]
Jang 2013	1,303	1,104.8	1609	1,323	1,049.9	1669	30.8%	-20.00 [-93.83, 53.83]
Murray 1999	375.3	236.1	26	284.7	136.2	19	19.1%	90.60 [-18.88, 200.08]
**Subtotal (95% CI)**			**1670**			**1736**	**100.0%**	**-4.08 [-61.55, 53.40]**
Heterogeneity: Tau² = 1370.52; Chi² = 4.23, df = 2 (P = 0.12); I² = 53%								
Test for overall effect: Z = 0.14 (P = 0.89)								
**5.1.2 Discharged LOS**								
Hsiao 2007	184.3	54.4	79	220	60.7	63	34.6%	-35.70 [-54.90, -16.50]
Jang 2013	285.7	178.7	3396	296.3	184.7	3286	39.3%	-10.60 [-19.32, -1.88]
Murray 1999	191.7	80.3	67	260.3	119.7	68	26.1%	-68.60 [-102.94, -34.26]
**Subtotal (95% CI)**			**3542**			**3417**	**100.0%**	**-34.42 [-63.68, -5.16]**
Heterogeneity: Tau² = 547.36; Chi² = 14.36, df = 2 (P = 0.0008); I² = 86%								
Test for overall effect: Z = 2.31 (P = 0.02)								

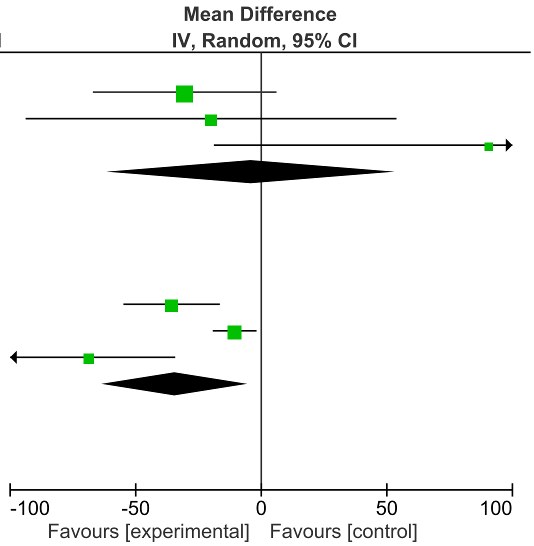

Test for subgroup differences: Chi² = 0.85, df = 1 (P = 0.36), I² = 0%

For peer review only

	POCT		Control		Risk Ratio
**Study or Subgroup**	**Events**	**Total**	**Events**	**Total**	**IV, Random, 95% CI**
**6.1.1 General Panel (ED)**					
Kendall 1998	55	859	48	867	1.16 [0.79, 1.68]
**6.1.2 Cardiac Panel (ED)**					
Goodacre 2011	6	1125	2	1118	2.98 [0.60, 14.74]
**6.1.3 Cardiac Panel (Out of hospital)**					
Ezekowitz 2014	4	240	5	240	0.80 [0.22, 2.94]

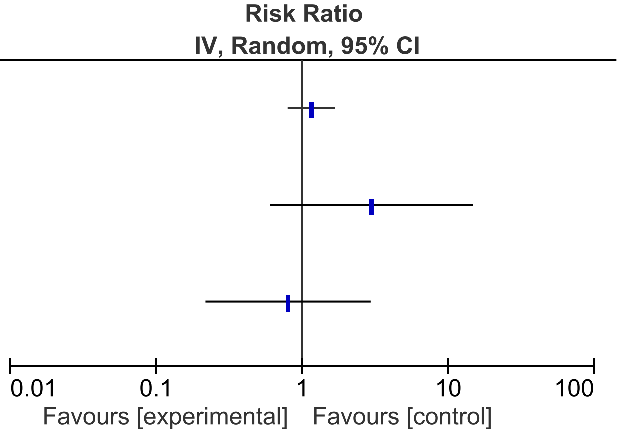

For peer review only

Appendix 1: Search Strategy

1	Ambulatory Care/
2	exp Ambulatory Care Facilities/
3	general practice/ or family practice/
4	general practitioners/ or physicians, family/ or physicians, primary care/
5	Primary Health Care/
6	Office Visits/
7	exp Emergency Service, Hospital/
8	Emergency Medical Services/
9	(ambulatory adj3 (care or setting? or facilit* or ward? or department? or service?)).ti,ab.
10	((general or family) adj2 (practi* or physician? or doctor?)).ti,ab.
11	(primary care or primary health care or primary healthcare).ti,ab.
12	(emergency adj3 (care or setting? or facilit* or ward? or department? or service?)).ti,ab.
13	(after hour? or afterhour? or "out of hour?" or ooh).ti,ab.
14	(clinic? or visit?).ti,ab.
15	((health* or medical) adj2 (center? or centre?)).ti,ab.
16	community health services/ or exp community health nursing/
17	Community Health Workers/
18	(community adj2 (health or health care or service? or program*)).ti,ab.
19	(community adj2 (worker? or aide? or volunteer? or assistant? or visitor?)).ti,ab.
20	((lay or volunteer) adj2 (health worker? or health aide? or health assistant?)).ti,ab.
21	((health* or medical) adj2 (facility or facilities)).ti,ab.
22	1 or 2 or 3 or 4 or 5 or 6 or 7 or 8 or 9 or 10 or 11 or 12 or 13 or 14 or 15
23	16 or 17 or 18 or 19 or 20 or 21
24	Point-of-Care Systems/
25	(("point of care" or POC) adj3 (test* or diagnos*)).ti,ab.
26	(("point of care" or POC) and (test* or diagnos*)).ti.

For peer review only

27	poct.ti,ab.
28	((rapid or bedside or bed-side or "near patient") adj3 (test* or diagnos*)).ti,ab.
29	((rapid or bedside or bed-side or "near patient") and (test* or diagnos*)).ti.
30	24 or 25 or 26 or 27 or 28 or 29
31	(istat or i-stat or afinion).ti,ab.
32	30 or 31
33	22 and 32
34	23 and 32
35	34 not 33

For peer review only

For peer review only - http://bmjopen.bmj.com/site/about/guidelines.xhtml

Appendix 2: Risk of bias assessment for Parvin et al, before-after study[27]

Selection		Performance	Detection	Attrition	Reporting	Other	Pre-specified confounders		
Random sequence generation	Allocation concealment	Blinding or participants/ personnel	Blinding of outcome assessment	Incomplete outcome data	Selective reporting		Baseline charactertisitics reported	Baseline charactersitic similar in intervention and control groups	Detailed description of usual care pathway
not randomised<br><br>**HIGH RISK**	not concealed; time periods of intervention and control described<br><br>**HIGH RISK**	none<br><br>**HIGH RISK**	not described<br><br>**UNCLEAR RISK**	not described<br><br>**UNCLEAR RISK**	not described<br><br>**UNCLEAR RISK**	control groups from before and after the intervention period; retrospective designation of presenting symptom codes<br><br>**HIGH RISK**	not reported<br><br>**HIGH RISK**	not reported<br><br>**HIGH RISK**	no details other than that samples are sent to central laboratoy via vacuum transport system<br><br>**HIGH RISK**

For peer review only

Appendix 3

Sensitivity Analysis

Figure 3: Disposition Decision (subgroups for patients who needed laboratory tests in addition to POCT)

Figure 3a) With Illahi[25]

Study or Subgroup	POC Mean	POC SD	POC Total	Lab Mean	Lab SD	Lab Total	Weight	Mean Difference IV, Random, 95% CI
**3.1.1 Patients who needed laboratory tests in addition to POCT**								
Lee 2011	48.5	22.7	616	88	27.5	609	95.3%	-39.50 [-42.33, -36.67]
Illahi 2012	137	59.3	18	166	67.61	29	0.6%	-29.00 [-65.82, 7.82]
**Subtotal (95% CI)**			**634**			**638**	**95.8%**	**-39.44 [-42.26, -36.62]**
Heterogeneity: Tau² = 0.00; Chi² = 0.31, df = 1 (P = 0.58); I² = 0%								
Test for overall effect: Z = 27.44 (P < 0.00001)								
**3.1.2 Patients whose blood work fell entirely within capabilities of POCT**								
Hsiao 2007	93.1	59.5	114	138.3	52.6	111	3.5%	-45.20 [-59.86, -30.54]
Illahi 2012	104.8	40.4	10	166	67.6	29	0.6%	-61.20 [-96.30, -26.10]
**Subtotal (95% CI)**			**124**			**140**	**4.2%**	**-47.58 [-61.11, -34.05]**
Heterogeneity: Tau² = 0.00; Chi² = 0.68, df = 1 (P = 0.41); I² = 0%								
Test for overall effect: Z = 6.89 (P < 0.00001)								
**Total (95% CI)**			**758**			**778**	**100.0%**	**-39.78 [-42.53, -37.02]**
Heterogeneity: Tau² = 0.00; Chi² = 2.32, df = 3 (P = 0.51); I² = 0%								
Test for overall effect: Z = 28.27 (P < 0.00001)								
Test for subgroup differences: Chi² = 1.33, df = 1 (P = 0.25), I² = 24.9%								

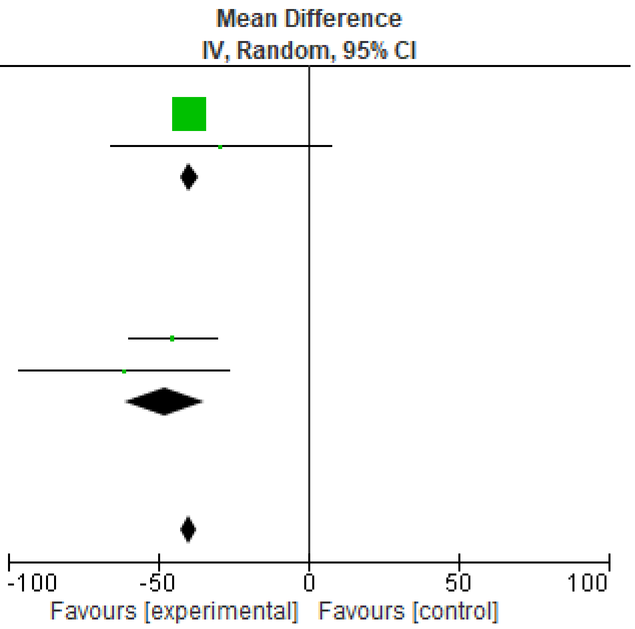

Figure 3b) Without Illahi[25]

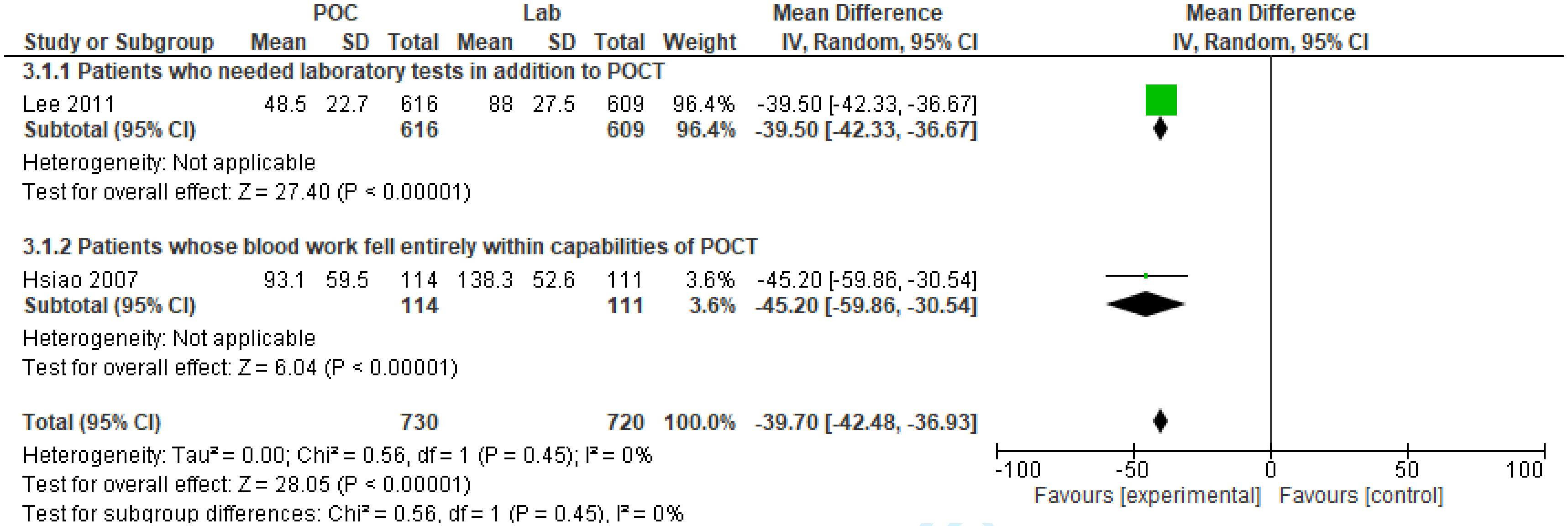

ew only

For peer review only

PRISMA 2009 Checklist

Section/topic	#	Checklist item	Reported on page #
**TITLE**			
Title	1	Identify the report as a systematic review, meta-analysis, or both.	1
**ABSTRACT**			
Structured summary	2	Provide a structured summary including, as applicable: background; objectives; data sources; study eligibility criteria, participants, and interventions; study appraisal and synthesis methods; results; limitations; conclusions and implications of key findings; systematic review registration number.	2
**INTRODUCTION**			
Rationale	3	Describe the rationale for the review in the context of what is already known.	4
Objectives	4	Provide an explicit statement of questions being addressed with reference to participants, interventions, comparisons, outcomes, and study design (PICOS).	4
**METHODS**			
Protocol and registration	5	Indicate if a review protocol exists, if and where it can be accessed (e.g., Web address), and, if available, provide registration information including registration number.	4
Eligibility criteria	6	Specify study characteristics (e.g., PICOS, length of follow-up) and report characteristics (e.g., years considered, language, publication status) used as criteria for eligibility, giving rationale.	5
Information sources	7	Describe all information sources (e.g., databases with dates of coverage, contact with study authors to identify additional studies) in the search and date last searched.	5
Search	8	Present full electronic search strategy for at least one database, including any limits used, such that it could be repeated.	Appendix A
Study selection	9	State the process for selecting studies (i.e., screening, eligibility, included in systematic review, and, if applicable, included in the meta-analysis).	5
Data collection process	10	Describe method of data extraction from reports (e.g., piloted forms, independently, in duplicate) and any processes for obtaining and confirming data from investigators.	5,6
Data items	11	List and define all variables for which data were sought (e.g., PICOS, funding sources) and any assumptions and simplifications made.	5
Risk of bias in individual studies	12	Describe methods used for assessing risk of bias of individual studies (including specification of whether this was done at the study or outcome level), and how this information is to be used in any data synthesis.	5,6
Summary measures	13	State the principal summary measures (e.g., risk ratio, difference in means).	6
Synthesis of results	14	Describe the methods of handling data and combining results of studies, if done, including measures of consistency (e.g., I^2) for each meta-analysis.	6

For peer review only - http://bmjopen.bmj.com/site/about/guidelines.xhtml

PRISMA 2009 Checklist

Section/topic	#	Checklist item	Reported on page #
Risk of bias across studies	15	Specify any assessment of risk of bias that may affect the cumulative evidence (e.g., publication bias, selective reporting within studies).	5,6
Additional analyses	16	Describe methods of additional analyses (e.g., sensitivity or subgroup analyses, meta-regression), if done, indicating which were pre-specified.	10
**RESULTS**			
Study selection	17	Give numbers of studies screened, assessed for eligibility, and included in the review, with reasons for exclusions at each stage, ideally with a flow diagram.	6
Study characteristics	18	For each study, present characteristics for which data were extracted (e.g., study size, PICOS, follow-up period) and provide the citations.	8
Risk of bias within studies	19	Present data on risk of bias of each study and, if available, any outcome level assessment (see item 12).	Figure 2, appendix 2
Results of individual studies	20	For all outcomes considered (benefits or harms), present, for each study: (a) simple summary data for each intervention group (b) effect estimates and confidence intervals, ideally with a forest plot.	Figures 4-6
Synthesis of results	21	Present results of each meta-analysis done, including confidence intervals and measures of consistency.	6-11
Risk of bias across studies	22	Present results of any assessment of risk of bias across studies (see Item 15).	Figure 2, appendix 2
Additional analysis	23	Give results of additional analyses, if done (e.g., sensitivity or subgroup analyses, meta-regression [see Item 16]).	Appendix 3
**DISCUSSION**			
Summary of evidence	24	Summarize the main findings including the strength of evidence for each main outcome; consider their relevance to key groups (e.g., healthcare providers, users, and policy makers).	11-13
Limitations	25	Discuss limitations at study and outcome level (e.g., risk of bias), and at review-level (e.g., incomplete retrieval of identified research, reporting bias).	3, 11-13
Conclusions	26	Provide a general interpretation of the results in the context of other evidence, and implications for future research.	11-13
**FUNDING**			
Funding	27	Describe sources of funding for the systematic review and other support (e.g., supply of data); role of funders for the systematic review.	14

For peer review only

PRISMA 2009 Checklist

From: Moher D, Liberati A, Tetzlaff J, Altman DG, The PRISMA Group (2009). Preferred Reporting Items for Systematic Reviews and Meta-Analyses: The PRISMA Statement. PLoS Med 6(7): e1000097. doi:10.1371/journal.pmed1000097

For more information, visit: **www.prisma-statement.org**.

For peer review only

For peer review only - http://bmjopen.bmj.com/site/about/guidelines.xhtml
